# An Injectable Enzymatically Crosslinked Carboxymethylated Pullulan/Chondroitin Sulfate Hydrogel for Cartilage Tissue Engineering

**DOI:** 10.1038/srep20014

**Published:** 2016-01-28

**Authors:** Feng Chen, Songrui Yu, Bing Liu, Yunzhou Ni, Chunyang Yu, Yue Su, Xinyuan Zhu, Xiaowei Yu, Yongfeng Zhou, Deyue Yan

**Affiliations:** 1 School of Chemistry and Chemical Engineering, State Key Laboratory of Metal Matrix Composites, Shanghai Jiao Tong University, 800 Dongchuan Road, Shanghai 200240, P. R. China; 2 Department of Orthopaedic Surgery, Shanghai Jiao Tong University Affiliated Sixth People’s Hospital, 600 Yishan Road, Shanghai, 200233, P. R. China; 3 Department of Oral and Maxillofacial Surgery, First Affiliated Hospital of Harbin Medical University, 23 Youzheng Street, Nangang District, Harbin, P. R. China; 4 Jiangsu Collaborative Innovation Center of Biomedical Functional Materials, Jiangsu Key Laboratory of Biomedical Materials, College of Chemistry and Materials Science, Nanjing Normal University, Nanjing 210046, P. R. China

## Abstract

In this study, an enzymatically cross-linked injectable and biodegradable hydrogel system comprising carboxymethyl pullulan-tyramine (CMP-TA) and chondroitin sulfate-tyramine (CS-TA) conjugates was successfully developed under physiological conditions in the presence of both horseradish peroxidase (HRP) and hydrogen peroxide (H_2_O_2_) for cartilage tissue engineering (CTTE). The HRP crosslinking method makes this injectable system feasible, minimally invasive and easily translatable for regenerative medicine applications. The physicochemical properties of the mechanically stable hydrogel system can be modulated by varying the weight ratio and concentration of polymer as well as the concentrations of crosslinking reagents. Additionally, the cellular behaviour of porcine auricular chondrocytes encapsulated into CMP-TA/CS-TA hydrogels demonstrates that the hydrogel system has a good cyto-compatibility. Specifically, compared to the CMP-TA hydrogel, these CMP-TA/CS-TA composite hydrogels have enhanced cell proliferation and increased cartilaginous ECM deposition, which significantly facilitate chondrogenesis. Furthermore, histological analysis indicates that the hydrogel system exhibits acceptable tissue compatibility by using a mouse subcutaneous implantation model. Overall, the novel injectable pullulan/chondroitin sulfate composite hydrogels presented here are expected to be useful biomaterial scaffold for regenerating cartilage tissue.

Cartilage tissue engineering (CTTE), which offers advantages over the current treatment strategies of damaged cartilage tissue, is proposed as an emerging promising therapeutic alternative for improved cartilage regeneration^1^,^2^,^3^. In native cartilage tissue, chondrocytes resident in a highly hydrated three dimensional (3D) extracellular matrix (ECM) environment in which chondrocytes secrete abundant matrix molecules such as collagen type II and glycosaminoglycans (GAGs). The GAGs, like chondroitin sulfate (CS), hyaluronic acid (HA) and heparin sulphate (HS), are usually attached to ECM proteins to form proteoglycans^2^,^4^. In addition to the physical cues of cartilaginous ECM, chondrocytes are not only exposed to an array of biological cues throughout the ECM that direct cellular behaviour, but also constantly interact with the surrounding ECM, which give rise to a dynamic transfer of information between extracellular and intracellular space^5^. Therefore, furthest imitating the characteristics of cartilaginous ECM is vital to the rational design of biomaterial scaffolds for CTTE^6^,^7^,^8^.

In general, an excellent CTTE scaffold, which is successfully applied to clinical cartilage repair, must have the following characteristics: i) be easy to handle, and be reliable and reproducible under physiological conditions; ii) be controllable and suitable physicochemical properties; iii) preferably mimic cartilaginous ECM features and promote chondrogenic potential of cells; iv) be fully biocompatible with cell growth and tissue remodelling; v) easily fill the defect sites, and strongly adhere and integrate with the surrounding native cartilage tissue^8^,^9^,^10^,^11^. Among a variety of biomaterial scaffolds for CTTE, injectable hydrogels consisting of natural polysaccharides are likely to fulfil these requirements, since they can not only provide a biocompatible, highly hydrated 3D environment similar to cartilaginous ECM, and facilitate transport of nutrients and cellular metabolites through the elastic network^12^,^13^,^14^,^15^, but also more effectively encapsulate and deliver desired cells and bioactive molecules to targeted sites of cartilage regeneration via a simple minimally invasive procedure, along with easy fill large and irregular complex defects^6^,^7^,^10^. More importantly, the natural polysaccharides, such as HA, pullulan, collagen, CS, gelatin, HS and chitosan, resemble the macromolecular structure of tissue ECM, and thus are able to mimic many features of tissue ECM and have the potential to direct cellular behaviour during tissue regeneration. Many of natural polysaccharide-based hydrogels demonstrated adequate biocompatibility and biodegradability which also made them an appropriate candidate for biomaterial scaffolds development^10^,^16^.

Until now, various crosslinking approaches, including physical and chemical crosslinking, have been developed to construct injectable hydrogels for cartilage regeneration^7^,^17^. Recently, an enzymatic crosslinking strategy using horseradish peroxidase (HRP) and hydrogen peroxide (H_2_O_2_) has paid increasing attention to the *in situ* formation of the hydrogels for biomedical applications due to its substrate specificity and efficiency, mild reaction conditions, tunable reaction rate and good cyto-compatibility^3^,^4^,^12^. Using the HRP-mediated crosslinking system, covalent bonds between hydroxyphenyl groups are efficiently formed. Thus, the phenol-conjugated polymers may covalently bind to the hydroxyphenyl residues of tyrosine containing in the ECM proteins during *in situ* gel formation, which can lead to better integrate with the surrounding native tissue^2^. Additionally, the consumption rate of H_2_O_2_ can be explicitly controlled by the modulation HRP concentration, thus the crosslinking approach enables independently tune gelation rate and mechanical properties of the hydrogels by varying the concentrations of HRP and H_2_O_2_, respectively. In particular, the advantages of the method for the crosslinking of natural polymers are able to circumvent harsh chemical conditions, and thus possible loss of bioactivity is avoided^17^. Therefore, in recent years, a variety of hydrogels have been developed taking advantage of this system for the crosslinking of phenol-conjugated polymers, including dextran^1^,^2^,^18^, HA^19^,^20^, alginate^21^, gelatin^22^,^23^, chitosan^24^, HS^3^, glycopolypeptide^25^, poly(ethylene glycol) (PEG)^13^,^14^, tetronic (a four-armed block copolymer of poly(ethylene oxide) and poly-(propylene oxide))^12^,^26^ and poly(L-glutamic acid)^15^ as excellent injectable biomaterials for multiple biomedical applications. Among them, dextran-tyramine hydrogels in combination with HA and HS have shown high potential as artificial ECM for CTTE. It has been demonstrated that the polysaccharide-based hydrogels are good cyto-compatible and capable of maintaining chondrocyte phenotype and promoting biosynthesis of cartilaginous ECM^2^,^3^,^19^.

Pullulan is biodegradable, and has high adhesion and good mechanical properties because of its unique linkage pattern^27^. It is also neutral, non-toxic, non-immunogenic and non-carcinogenic, along with Food and Drug Administration (FDA) approved for a variety of applications^28^,^29^. Thus, it has been widely explored recently for various biomedical applications, such as drug and gene delivery^30^ and tissue engineering^31^,^32^,^33^,^34^,^35^. For example, Ali Khademhosseini *et al*. successfully prepared methacrylated pullulan/methacrylated gelatin hydrogel by exposure to ultraviolet (UV) light in the presence of photo-initiator for creating cell-responsive microscale tissues^28^. Recently, Catherine Le Visage and Joëlle Amédée *et al*. found the pullulan/dextran porous scaffolds cross-linked with trisodium trimetaphosphate (STMP) were able to promote vascular regeneration, heart tissue repair and bone tissue regeneration^33^,^34^,^36^,^37^,^38^. Additionally, Geoffrey C. Gurtner *et al*. used the biomimetic pullulan/collagen scaffolds by STMP crosslinking to accelerate normal wound healing^31^,^35^. In particular, more recently, Frank Barry *et al*. reported the culture of mesenchymal stem cells (MSCs) with pullulan-treated medium, and found pullulan positively affected MSC osteogenic differentiation and sustained chondrogenic potential. More importantly, pullulan dramatically enhanced MSCs retention on the fibrillated surface of osteoarthritic articular cartilage owing to its effective bio-adhesive^39^. However, to the best of our knowledge, the pullulan-based injectable hydrogel as a CTTE scaffold has not been previously reported.

There are several difficulties lying for the pullulan-based injectable hydrogel as a CTTE scaffold. Firstly, as mentioned above, the common crosslinking methods for pullulan include UV light irradiation and STMP performed at 40 and 50 °C under alkaline conditions^27^,^28^. These crosslinking conditions are suitable to prepare bulk materials but they may be too harsh for the preparation of *in situ* injectable hydrogels. Secondly, pullulan exhibited the limited chondrogenic potential of MSCs^39^. Thus, the pure pullulan-based hydrogel might not perfectly mimic cartilaginous ECM microenvironment, probably owing to the absence of chondro-inductive biochemical cues. So, some much milder crosslinking method and multifunctional components are very important for the preparation of high-performance pullulan-based injectable hydrogel.

Herein, to our knowledge, we report for the first time on the pullulan-based multicomponent injectable hydrogel as a CTTE scaffold. The HRP-mediated crosslinking strategy was applied in our hydrogel system, thus the crosslinking process can be performed in physiological conditions. In addition, since CS has a number of attractively biological features for CTTE including anti-inflammatory activity, chondro-protective properties, improved wound healing and biological activity at the cellular level that may help to restore arthritic joint function^40^,^41^,^42^, the bioactive molecule was also incorporated into our hydrogel system to better recapitulate native cartilage microenvironment^5^,^41^,^42^,^43^,^44^. The obtained pullulan/CS injectable hydrogel has displayed the properties of fast gelation rate, adjustable mechanical properties, controllable degradation behaviour, excellent cyto-compatibility and tolerable tissue compatibility. More importantly, the composite hydrogel provided a host tissue-mimetic environment for maintaining chondrocyte phenotype and enhancing chondrogenesis.

## Results and Discussion

### Synthesis and characterization of CMP-TA and CS-TA conjugates

**Figure 1:**
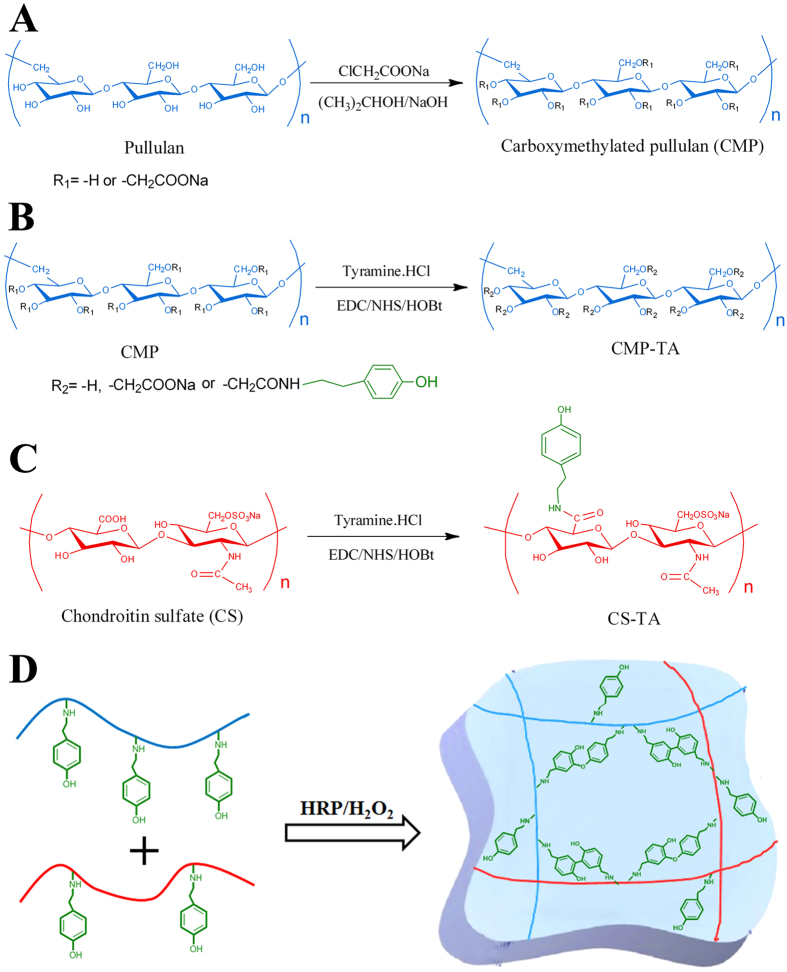
Synthesis of CMP, CMP-TA and CS-TA (**A–C**). (**D**) Hydrogel formation from CMP-TAs and CS-TAs via HRP-mediated crosslinking in the presence of H_2_O_2_.

CMP was synthesized as previously reported^45^ and had a degree of substitution with carboxymethyl groups of about 10% (determined by conductimetric titration), i.e. 0.3 of the three hydroxyl groups of anhydroglucose unit was transformed into carboxylate groups (Fig. 1A). The key materials of this study, the CMP-TA and CS-TA conjugates were successfully synthesized by the coupling reaction of the amino groups of TA to the carboxylic acid groups of CMP and CS using EDC/NHS activation (Figs 1B and C). ^1^H NMR results showed that the DS of CMP-TA and CS-TA were about 6.0 and 7.0, respectively (Figs S1A and B). Additionally, the average molecular weights of CMP-TA and CS-TA were approximately 7.4 × 10^4^ and 6.3 × 10^4^ by GPC measurements, respectively.

### Formation and characterization of CMP-TA/CS-TA hydrogel

In the study, CMP-TA/CS-TA hydrogels were rapidly formed by simply mixing CMP-TA and CS-TA conjugates with HRP and H_2_O_2_ in PBS under mild conditions, which was a highly efficient method to prepare *in situ* forming hydrogel. The mechanisms for the biochemical reaction enabling covalent crosslinking of phenol derivatives previously have been discussed in the literature^20^,^23^. Briefly, the TA moieties of CMP-TA and CS-TA were conjugated with each other through a HRP-mediated oxidative reaction via the carbon-carbon bond at the ortho positions or via the carbon-oxygen bond between the carbon at the ortho position and the phenoxy oxygen (Fig. 1D). Additionally, the internal microstructure of CMP-TA/CS-TA hydrogels displayed a heterogeneous, continuous and porous microstructure by virtue of the freeze-drying step, with the pores being the result of ice crystal formation, resembling other natural macromolecular hydrogel system structures (Fig. S2). The pore diameter is in the range of 50–150 μm, and it increases with the increase of CS-TA contents, which is probably attributed to the decease of crosslinking dendity with more CS-TAs.

**Figure 2:**
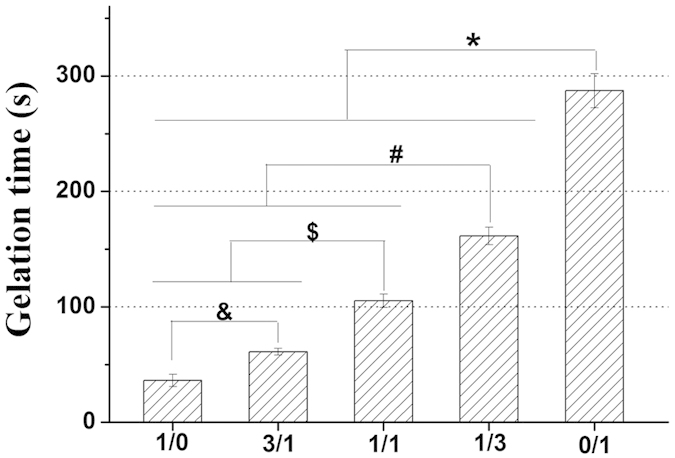
Gelation time of CMP-TA/CS-TA hydrogels as a function of CMP-TA/CS-TA weight ratio, and the final concentrations of polymer, HRP and H_2_O_2_ were kept at 5 wt. %, 0.6 units/mL and 1 mM, respectively (n = 8, p < 0.01).

Gelation rate is an important factor to consider in the development of injectable hydrogels for CTTE, especially where the application is related to cells and bioactive molecules encapsulation and delivering^14^. Thus, the crosslinking reaction was optimized with respect to the suitable gelation time. In this study, gelation time as a function of CMP-TA/CS-TA weight ratio was presented in Fig. 2. When weight ratio of CMP-TA/CS-TA decreased from 1/0 to 0/1, the gelation time significantly increased from 36 s to 287 s (p < 0.01) under the final concentrations of 0.6 unit/mL HRP and 1 mM H_2_O_2_. The increase in gelation time of the hydrogels containing CS-TA compared to the CMP-TA hydrogel might be explained by the unfavorable interactions with the active site of the enzyme caused by the more acid groups in CS chains (e.g. 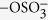 and 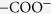) either through steric hindrance or charge interactions^3^. Additionally, the use of a low H_2_O_2_ concentration was required because the remaining H_2_O_2_ could induce cytotoxicity, although most of H_2_O_2_ was decomposed via the enzymatic reaction^26^. Kurisawa *et al*. previously reported that the hyaluronic acid-tyramine hydrogel was biocompatible and no cytotoxicity in the presence of 70 mM H_2_O_2_^20^. Thus, in our crosslinking system, the hydrogels based on CMP-TA/CS-TA have been developed by using relatively low amounts of H_2_O_2_, which may avoid toxic effects of H_2_O_2_ at high concentrations. In addition, the effect of the concentrations of polymer, HRP and H_2_O_2_ on the gelation time of CMP-TA/CS-TA hydrogel was discussed in detail (Fig. S3). From the above results it was found that the gelation time of CMP-TA/CS-TA hydrogel could be easily controlled by adjusting the weight ratio of polymer as well as the concentrations of polymer and HRP, which made the system highly suitable as injectable hydrogel for CTTE. In particular, a controlled gelation rate and a low concentration of hydrogel precursor solution have various advantages for CTTE application. For example, an adequate gelation rate is essential to prevent diffusion of the precursors and fill any shape defect site of cartilage. A relatively low concentration of hydrogel precursor is easy to handle and capable of loading with cells and/or bioactive molecules homogeneously.

**Figure 3:**
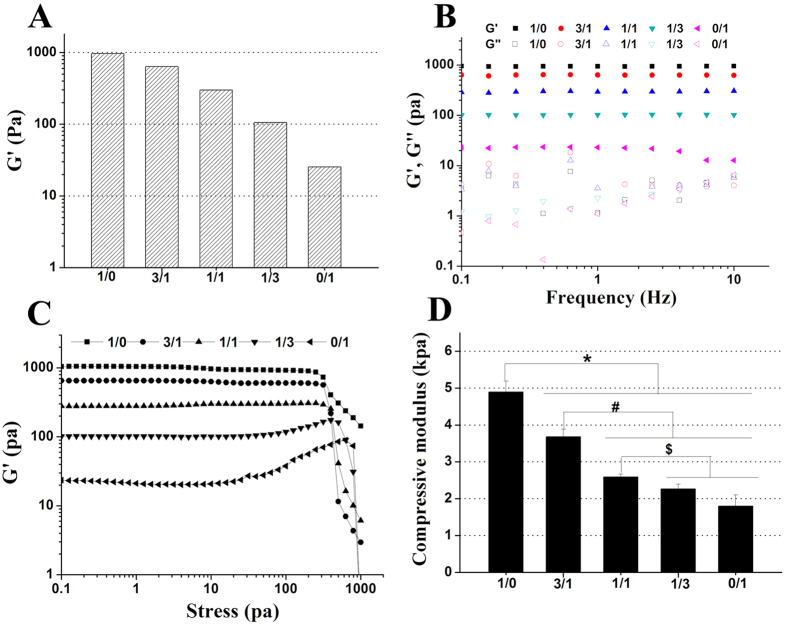
Storage moduli (G’) (**A**), Frequency sweep (**B**), Stress sweep (**C**) and Compressive modulus (n = 3, p < 0.01) (**D**) of CMP-TA/CS-TA hydrogels as a function of CMP-TA/CS-TA weight ratio, and the final concentrations of polymer, HRP and H_2_O_2_ were kept at 5 wt. %, 0.6 units/mL and 1 mM, respectively.

For successful clinical application, the mechanical properties of an injectable hydrogel are very important. For instance, the hydrogel in the defect should possess suitable mechanical strength to withstand biomechanical loading and provide temporary support for the cells. If not, any nascent tissue formation will probably fail due to excessive deformation^1^. For this purpose, we studied particularly the mechanical properties of CMP-TA/CS-TA hydrogel by rheological and compressive modulus measurements. Initially, rheological measurements were performed to study the influence of the different weight ratios of CMP-TA/CS-TA on the viscoelastic properties of hydrogels (Fig. 3). The CMP-TA hydrogel (5 wt. %) showed a high G′ value of 963 Pa at fixed HRP concentration of 0.6 unit/mL and H_2_O_2_ concentration of 1 mM. Moreover, the G’ values of CMP-TA/CS-TA hydrogel decreased from 963 to 25 Pa with decreasing weight ratio in the time sweeps (Fig. 3A). Based on the results of the DS of CMP-TA and CS-TA (Fig. S1A,B), we deduced that the TA concentration in CMP-TA solution was about double of the TA concentration in CS-TA solution at the same polymer concentration. Therefore, the high G′ of the CMP-TA hydrogel could be attributed to either the higher crosslinking density or the higher stiffness of the polysaccharide itself^3^. For frequency sweep, except for the weakest hydrogel (0/1), the G′ values of the rest of hydrogels were frequency independent with in a range of 0.1–10 Hz, indicating rigid and elastic networks (Fig. 3B). Additionally, the G’ values are always larger than the loss modulus (G″) values indicating that the hydrogels display a predominantly elastic behavior (Fig. 3B, Figs S4C, S5C and S6C). In general, the breakdown of hydrogel structure, denoted by a rapid decline of G’, marked the end of linear viscoelastic region at the critical stress^46^. The stress sweep tests showed that the rest of hydrogels were quite robust except for the weakest hydrogel (0/1) (Fig. 3C). Notably, the fracture stress (the critical stress) decreased with increasing the CMP-TA content. The observed result was most likely ascribed to the inherent brittle structure of the hydrogel possessing high G'^46^. Additionally, the effect of the concentrations of polymer, HRP and H_2_O_2_ on the rheological behaviour of CMP-TA/CS-TA hydrogel was expounded detailedly (Figs S4, S5 and S6). Subsequently, the factors including polymer weight ratio as well as the concentrations of polymer, HRP and H_2_O_2_, affected the compressive modulus of hydrogel were also investigated (Figs 3D, S4E, S5E and S6E). Of note, the effect of these factors on the compressive modulus was coincident with the G’ in the time sweeps (Figs 3A, S4A, S5A and S6A). Taken together, the above results clearly indicated that the mechanical properties of our injectable hydrogel system could be easily regulated by varying concentrations of polymer and H_2_O_2_ as well as weight ratio of polymer whilst still maintaining the suitable gelation rate. The mechanism of the independent tuning of gelation rate and mechanical properties of the hydrogels has been explained in detail^13^,^20^. Thus, we concluded that the independent tuning achieved in CMP-TA /CS-TA hydrogel was due to the catalytic reaction of HRP and H_2_O_2_ and would be useful for improved cartilage defect repair.

**Figure 4:**
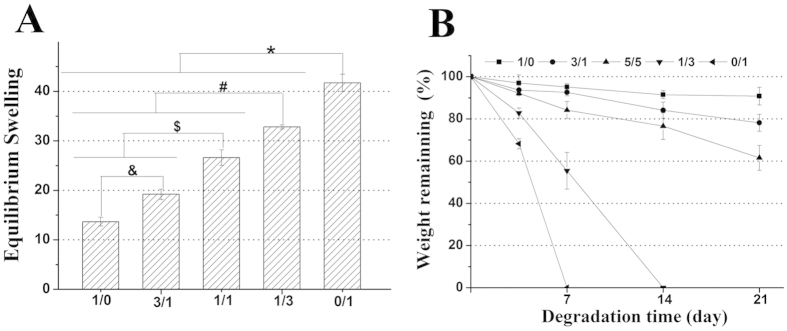
(**A**) The equilibrium swelling ratio (ESR) of CMP-TA/CS-TA hydrogels as a function of CMP-TA/CS TA weight ratio, and the final concentrations of polymer, HRP and H_2_O_2_ were kept at 5 wt. % , 4.8 units/mL and 5 mM, respectively (n = 6, p < 0.01). (**B**) Degradation of CMP-TA/CS-TA hydrogel as a function of CMP-TA/CS-TA weight ratio, and the final concentrations of polymer, HRP and H_2_O_2_ were kept at 5 wt. %, 4.8 units/mL and 5 mM, respectively (n = 6).

For this study, the equilibrium swelling ratio (ESR) behaviour of CMP-TA/CS-TA hydrogels formed with various polymer compositions and the concentrations of HRP and H_2_O_2_ was determined in PBS for 36 h (Figs 4A, S7A and B). Additionally, *in vitro* the degradation behaviour of the hydrogels formed with various polymer compositions and H_2_O_2_ concentration was investigated in PBS for predetermined time intervals (Figs 4B and S7C). The ESR and the degradation rate of the hydrogels (5 wt. %) formed with the different weight ratios of CMP-TA/CS-TA at fixed HRP concentration of 4.8 unit/mL and H_2_O_2_ concentration of 5 mM were depicted in Figs 4A and B, respectively. The ESR of all hydrogels ranged from 14 to 42. Notably, the high ESR may allow cells to exchange easily nutrients/metabolites, and also promote signal transduction and message communication^1^,^3^. Additionally, these CMP-TA/CS-TA hydrogels (1/0, 3/1 and 1/1) were rather stable as compared to the hydrogels (1/3 and 0/1). After 21 day of degradation, the weight remaining ratios of these hydrogel were about 91%, 78% and 62%, respectively. However, the hydrogels prepared with the CMP-TA/CS-TA weight ratios of 1/3 and 0/1 were completely degraded after 7 and 14 days, respectively. The hydrogels showed a general trend of a lower ESR and slower degradation rate that correlated with hydrogels of a higher cross-link density and mechanical strength^3^,^26^. As expect, by increasing CS-TA content in the hydrogels, the corresponding ESR values significantly increased (p < 0.01) and the degradation rate remarkably accelerated in PBS. In addition, as shown in Table S2, the ESR values of the gels show a similar chaning trend when placed in pure water and tissue fluid-like solution (Hanks’ Balanced Salt Solution, H9394, Sigma). The high ESR and the rapid degradation rate of the hydrogels containing CS-TA could be either due to the lower crosslinking density or due to the higher electrostatic repulsion caused by negatively charged CS-TA chains at pH 7.4^13^. In summary, our findings suggested that the ESR and the degradation rate of the hydrogel could be readily controlled by varying polymer weight ratio and H_2_O_2_ concentration. These results would provide practical, valuable information in attempts to achieve optimal CTTE, where controlling degradability of the hydrogels was critical.

### *In vitro* biological analysis of encapsulated chondrocytes in the hydrogels

In the study, the primary chondrocytes were encapsulated into CMP-TA/CS-TA hydrogels (5 wt. %) at fixed HRP concentration (4.8 unit/mL) and H_2_O_2_ concentration (5 mM), and incubated in the chondrocyte specific medium containing basic fibroblast growth factor (bFGF, 5 ng/mL) for 14 days. The CMP-TA/CS-TA hydrogels at different weigh ratios of 1/0, 3/1 and 1/1 were chosen for the biological studies. Moreover, the three hydrogels were abbreviated respectively, as CC-1, CC-2 and CC-3. Then, the impacts of the three different types of hydrogels on chondrocyte behaviour were further investigated by the biological analysis for Live-Dead assay, DNA content, RT-PCR, Immunofluorescent staining and WB as well as total collagen content.

**Figure 5:**
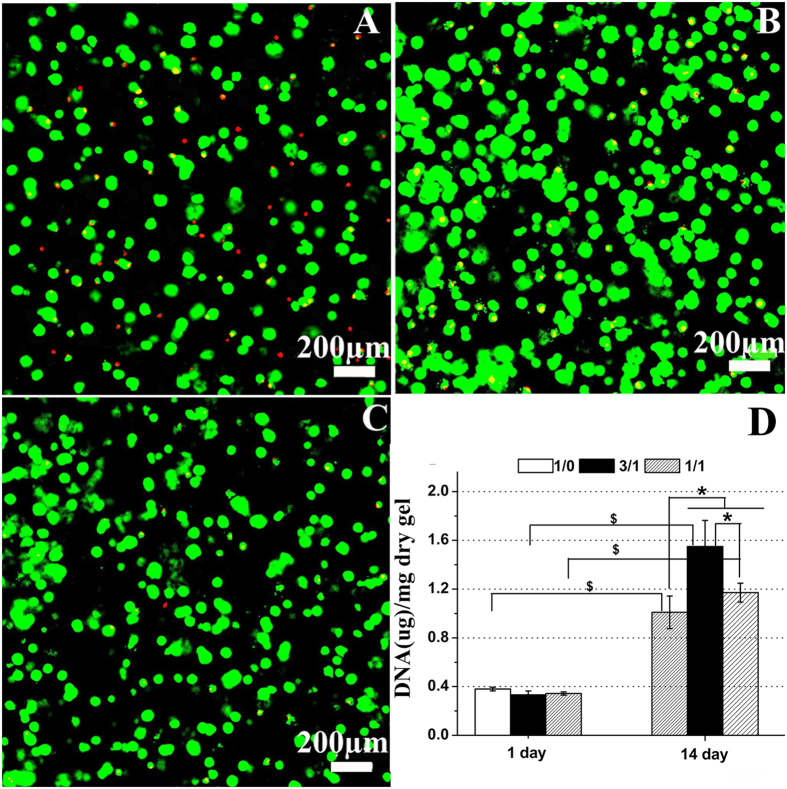
Live–dead assay showing chondrocytes encapsulated in CMP-TA/CS-TA hydrogels as a function of CMP-TA/CS-TA weight ratio of (**A**) 1/0, (**B**) 3/1 and (**C**) 1/1. The DNA content of CMP-TA/CS-TA hydrogels containing chondrocytes after culturing for 14 days (**D**) (n = 4, p < 0.05).

Prior to evaluating the chondrogenic potential of encapsulated chondrocytes, it is critical to ensure that the cells survive the hydrogel formation process and are viable throughout the duration of the culture period^47^. In present study, representative cell viability images from Live-Dead staining qualitatively showed cell distribution and density over 14 days of culture period (Figs 5A-C). About 15-20% dead cells were present in the CC-1 hydrogel, while over 95% chondrocytes remained viable in the CC-2 and CC-3 hydrogels. Overall, a large number and high percentage of viable cells were evident and distributed relatively homogeneously within the three types of hydrogels, indicating that these hydrogels using the enzyme crosslinking conditions were excellently cyto-compatible for chondrocytes. The above preliminary studies suggested that the incorporation of CS in the hydrogels was more conducive to cell viability and proliferation. These findings were further confirmed with DNA content measurement. Next, chondrocytes proliferation was monitored by quantifying DNA content of the hydrogels over the culture period at day1 and day 14 (Fig. 5D). DNA content was expressed as DNA amount normalized to the dry weight of remaining gel. Overall, DNA content significantly depended on time and hydrogel composition (p < 0.05). Initially, DNA values (p > 0.05) showed similar numbers of cells in the three hydrogels after 1 day of encapsulation, suggesting comparable encapsulation efficiencies in the hydrogels. Further, DNA contents in the CC-1, CC-2 and CC-3 hydrogels at day 14 were about 2.63, 4.70 and 3.44 times higher than their values at day 1, respectively (p < 0.05), indicative of chondrocyte proliferation. Specifically, at day 14, DNA contents in the CC-2 and CC-3 hydrogels were about 1.55 and 1.17 times higher than that in the CC-1 hydrogel, respectively (p < 0.05), which clearly demonstrated that the hydrogels containing CS-TA were more beneficial to chondrocyte proliferation compared with the CMP-TA hydrogel. Interestingly, the result was in accordance with previous studies demonstrating that incorporating CS within the biomaterial scaffolds facilitated cell proliferation^16^,^47^,^48^,^49^. Overall, the relatively high cell viability and proliferation observed for the hydrogels containing CS-TA could be ascribed to both the enhancement of nutrient exchange in these highly swollen hydrogels (Fig. 4A) and the potential biological roles of CS. In particular, several previous studies have testified that the different contents of CS within biomaterial scaffolds have significantly influenced on cell proliferation, differentiation and biosynthesis of cartilaginous ECM^16^,^41^,^43^,^47^,^50^. Therefore, we daringly presumed here that, the different contents of CS-TA in the hydrogels also had remarkably impacted on chondrocyte behaviour, furthermore, the CC-2 hydrogel provided a best host tissue mimetic microenvironment for maintaining chondrocyte phenotype and enhancing cartilaginous ECM deposition compared with the others *in vitro*.

**Figure 6:**
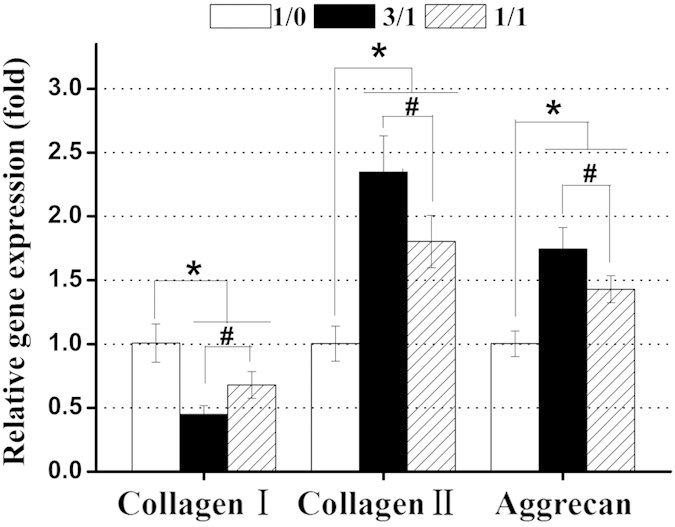
Quantitative RT-PCR of collagen type I, collagen type II and aggrecan by encapsulating chondrocytes into CMP-TA/CS-TA hydrogels after culturing for 14 days. The gene expression of these markers was normalized to expression of the housekeeping gene β-actin (n = 3, p < 0.05).

**Figure 7:**
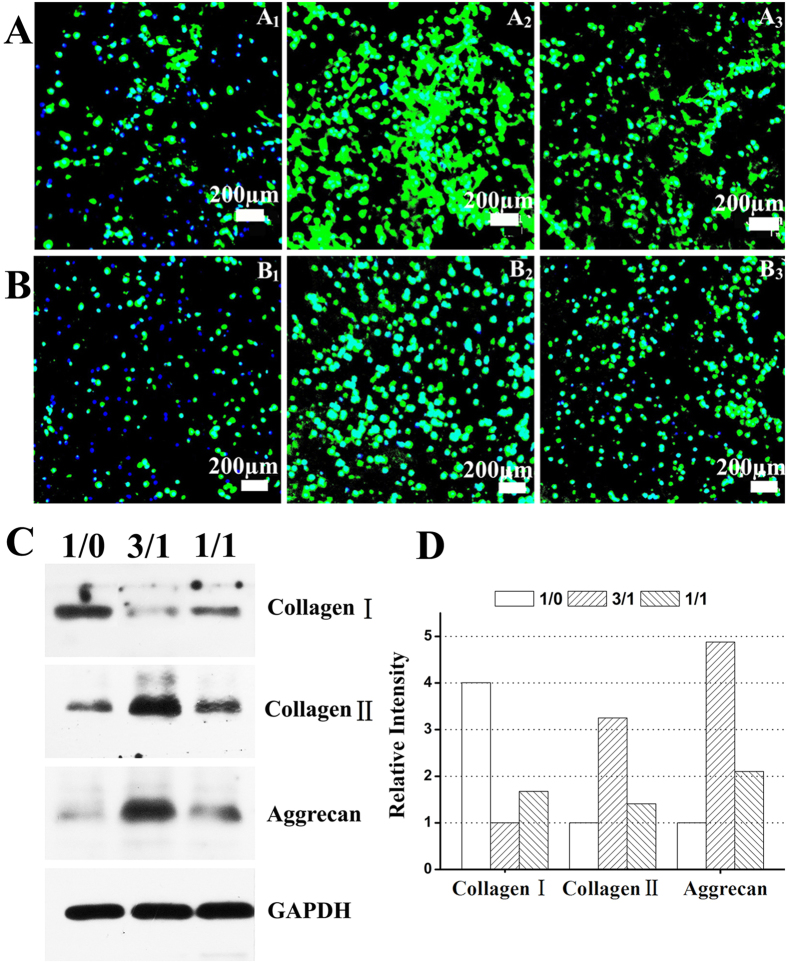
Collagen type II (**A**), aggrecan (**B**) immunofluorescent staining and WB analysis (**C**,**D**) of CMP-TA/CS TA hydrogels as a function of CMP-TA/CS-TA weight ratio of 1/0 (A_1_, B_1_), 3/1 (A_2_, B_2_) and 1/1 (A_3_, B_3_) containing chondrocytes after culturing for 14 days.

**Figure 8:**
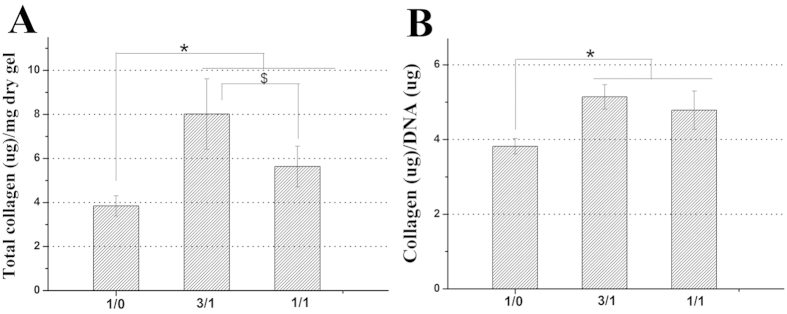
Total collagen in CMP-TA/CS-TA hydrogels containing chondrocytes after culturing for 14 days (n = 4, p < 0.05) (**A**,**B**).

To further substantiate our hypothesis, we evaluated gene and protein expression levels of collagen type I (dedifferentiation marker), collagen type II and aggrecan (chondrogenic marker) as well as accumulation of total collagen in the hydrogels. In current study, the changes in gene and protein expression levels of collagen type I,collagen type II and aggrecan in response to stimulation with the three types of hydrogels were determined using quantitative RT-PCR, immunofluorescent staining and WB analysis (Figs 6 and 7). Interestingly, the gene and protein expression levels of these markers were also significantly dependent on the hydrogel composition. Initially, as shown in Fig. 6, collagen type I gene expression of chondrocytes encapsulated in the CC-2 and CC-3 hydrogel was down regulated about 2.23 (p < 0.05) and about 1.47 (p < 0.05) times compared to that in the CC-1 hydrogel after 14 days of culture, respectively. In contrast, the chondrocytes encapsulated in the CC-2 and CC-3 hydrogels expressed about 2.35 (p < 0.05) and about 1.80 (p < 0.05) times up regulation gene expression of collagen type II compared to those in the CC-1 hydrogel, respectively. Similarly, aggrecan gene expression of chondrocytes encapsulated in the CC-2 and CC-3 hydrogels was up regulated about 1.74 (p < 0.05) and about 1.43 (p < 0.05) times compared to that in the CC-1 hydrogel, respectively. Additionally, in immunofluorescent staining images, cell nuclei fluoresced blue and collagen type II and aggrecan fluoresced green (Figs 7A and B). Many chondrocytes encapsulated in the CC-1 hydrogel did not show positive staining for collagen type II and aggrecan, again, the staining was only observed in the pericellular regions, with very little immunoreactivity in the regions between cells, which exhibited limited staining for these chondrogenic marker proteins. In contrast, a large number of chondrocytes encapsulated in the CC-2 and CC-3 hydrogels showed more intense positive staining for these chondrogenic marker proteins. Specifically, the maximum staining for collagen type II and aggrecan was observed in the CC-2 cell-laden construct. In addition, the expression levels of these marker proteins were clearly expressed on WB bands and appeared obvious differences among the three types of cell-laden constructs (Figs 7C and D). It was noteworthy that WB analysis and immunofluorescent staining results were in line with quantitative RT-PCR findings. Interestingly, the results correlated well with previous studies that the incorporation of CS into the matrixes were found to up regulate gene and protein expression levels of collagen type II and aggrecan in a content dependent manner^5^,^41^,^49^. In addition, the accumulation of total collagen was quantitatively examined by hydroxyproline assay, and the values were normalized to the corresponding dry gel weight or total DNA content of each sample. The collagen content in the CC-2 cell-laden construct was about 1.4 times higher than in the CC-3 cell-laden construct (p < 0.05), and about 2.1 times larger than in the CC-1cell-laden construct (p < 0.05) (Fig. 8A). Additionally, the normalized collagen contents were not significantly different between the CC-2 and CC-3 cell-laden constructs, although they were both significantly higher than the CC-1 cell-laden construct, by 1.35 and 1.25 times, respectively (p < 0.05) (Fig. 8B). Based on the above results, as expected, lower expression level of collagen type I and higher expression levels of collagen type II and aggrecan as well as total collagen accumulation were observed in cell-laden constructs containing CS-TA, indicating that CS-mediated microenvironment could well maintain chondrocyte phenotype and increase biosynthesis of cartilaginous ECM.

Pullulan has been combined with other biomaterials such as collagen and dextran for tissue engineering^31^,^33^,^34^,^35^. However, this study showed that the CMP-TA hydrogel permitted chondrogenic differentiation of chondrocytes by providing a 3D support, and this permissive environment only enabled chondrocytes to secrete fewer cartilaginous ECM *in vitro*. That is to say, it did not completely support chondrogenesis of chondrocytes. Interestingly, the result similar to previous report that pullulan only maintained chondrogenic potential of MSCs^39^. It was possible that, the CMP-TA molecule lacked the necessary biological cues to direct lineage-specific differentiation of primary chondrocytes.

The exact reasons of the improved chondrogenic potential of chondrocytes in the hydrogels containing CS-TA was currently unknown, but was more likely to be a combination of events. Initially, CS can be enzymatically degraded by cellular secretion of chondroitinase^5^. Thus, we inferred that the hydrogels containing the highly negatively charged CS-TA were beneficial to the increase in hydrogel swelling ratio and simultaneously underwent specifically enzymatic degradation in response to the cellular processes. This might result in a gel network with appropriate crosslinking density which allowed better transportation of nutrients to encapsulated chondrocytes. Though the precise biological roles of CS-TA in creating chondro-inductive microenvironments for encapsulated chondrocytes were not very clear, we speculated here that the incorporation CS-TA into the hydrogels might provide an opportunity to exploit the several bio-characteristics of CS derivative including the binding and modulation of proteins, the reaction with cartilaginous ECM and the direct participation in cellular activity.^43^ In native cartilage tissue, CS proteoglycans especially are known to play important roles in sequestration and signaling of positively charged growth factors *in vivo*, particularly during chondrogenesis^51^. In addition, previous studies have shown that CS interacts with various growth factors and that this binding is strictly controlled by the unique molecular structure and sulfation of CS^49^. Moreover, the growth factor interactions increase the half-life of growth factors and provide prolonged activity *in vivo*^41^. In the study, the immobilization of highly negatively charged CS-TA chains was in close proximity by crosslinking the hydrogels. In fact, this multivalent interaction maybe more comparable to CS proteoglycans, which are the native form that GAGs typically sequester proteins *in vivo*, with GAGs covalently linked to a protein core and anchored in close proximity^49^. Therefore, we inferred that the sulfate domains of CS-TA in the hydrogels was able to sequester growth factors supplemented in the chondrocyte specific medium or secreted by chondrocytes such as bFGF *in vitro*, and thus regulated their local concentration and activated the required signaling. This might facilitate chondrogenesis of encapsulated chondrocytes, and organization and remodeling of cartilaginous ECM. In support of this hypothesis, similar to previous reports that the CS participant systems have shown to promote chondrogenesis of cells in a manner that has primarily been attributed to their ability to bind growth factors^41^,^42^,^49^. Of note, as mentioned above, the CC-2 hydrogel enhanced the chondrogenic potential of encapsulated chondrocytes over the CC-3 hydrogel. This result might be partly due to the fact that the high negative charge (sulfate group) density of the CC-3 hydrogels could potentially decrease growth factors (such as bFGF) activity or inhibit transport within the hydrogel network relative to the moderate negative charge density of the CC-2 hydrogel. Meanwhile, the highly negatively charged CC-3 hydrogel might also prevent transport of other cell-secreted signals within the hydrogel, effectively inhibiting intercellular communication, which plays important roles in maintaining chondrocyte phenotype and supporting chondrogenic differentiation^51^. In addition, it is well-known that primary chondrocyte is highly sensitive for the changes of its surrounding microenvironment^10^. Therefore, it was important to consider that the incorporation CS-TA into the hydrogels might alter the extracellular microenvironment through related differences in osmolality^49^,^50^,^52^ or through various interactions with cartilaginous ECM, cell surface receptors, or other signaling molecules to influence chondrogenic potential of chondrocytes. For example, when the osmolality was altered by adding sodium ions to the culture medium of chondrocytes, the synthesis of cartilaginous ECM was affected in a concentration dependent manner^53^. Specifically, maximum synthesis was observed near physiological osmolality while the above or below physiological range synthesis decreased^50^. Extending the above findings to the present study, the different density of fixed negative charge in the hydrogels might attract free cations from the culture medium, resulting in the different osmolality within the hydrogels. The osmolality in CC-2 hydrogel might be closest to physiological level of osmolality compare to those of the other groups under the *in vitro* culture conditions. Additionally, to fully elucidate the biological roles of CS-TA in promoting chondrogenic potential, further investigation would be required to determine CS-TA molecule interactions with chondrocytes and cartilaginous ECM. Irrespective of the mechanism of action, further studies should validate whether the *in vitro* results shown here can be translated to enhanced quality of repair tissue *in vivo* in our future work.

### *In vivo* biocompatibility of the hydrogels

**Figure 9:**
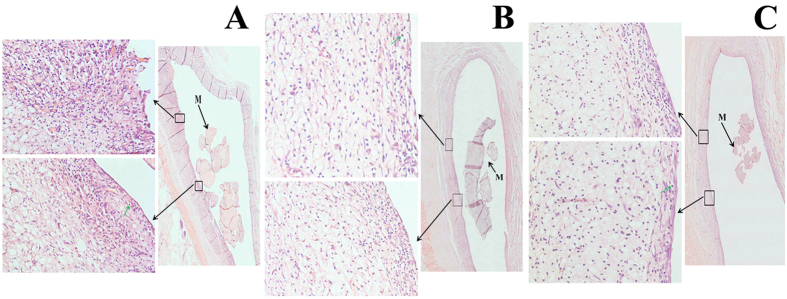
The implanted CMP-TA/CS-TA hydrogels of 1/0 (**A**), 3/1 (**B**) and 1/1 (**C**) on the backside of SD rats: the corresponding H&E staining was analyzed after subcutaneous implanting for 4 weeks. M represented residue of the implant and green arrow pointed to inflammatory cells.

In order to confirm the tissue compatibility of the hydrogels, the three different types of hydrogels in the absence of chondrocytes were implanted subcutaneously into SD rat model. After 4 weeks, tissue responses around the implanted hydrogels led to the development of fibrous tissue, and no macroscopic signs of inflammation or toxicity were evident in the tissue surrounding of the implants (Figs S8A-C). Interestingly, the formation of fibrous layers over implants *in vivo* has been frequently reported for a variety of feasible biomaterials^12^. Additionally, the inflammatory responses to the implanted hydrogels were studied by H&E staining of the surrounding tissues of the implants (Figs 9A-C). H&E staining results further confirmed that the implants were surrounded by fibrous capsules and had not yet completely degraded. It was noteworthy that an elevated number of inflammatory cells were observed in the all inplanted hydrogels, indicating that the three types of inplants had an mild inflammatory response. Moreover, there were not significant differences among in the three types of implants. Additionally, during our experiment, neither obvious tissue necrosis, edema, hyperemia and hemorrhaging nor muscle damage was observed. Therefore, the findings suggested that the CMP-TA/CS-TA hydrogel exhibited acceptable tissue compatibility *in vivo*, indicating that the hydrogel has potential as a CTTE scaffold for cartilage repair.

## Conclusion

In the current study, the injectable hydrogels were obtained by enzymatic crosslinking of CMP-TA and CS-TA conjugates under physiological conditions using HRP as a catalyst and H_2_O_2_ as an oxidant. The physicochemical properties of the hydrogel system were easily and reliably adjusted by changing the weight ratio and concentration of polymer as well as the concentrations of HRP and H_2_O_2_. When chondrocytes were encapsulated into CMP-TA/CS-TA hydrogels, it was found that cellular functions of chondrocytes, including cell viability and proliferation, gene and protein expression levels of these markers (collagen type I, collagen type II and aggrecan) as well as accumulation of synthesis ECM (total collagen), were remarkably affected by the content of CS-TA in the hydrogels. Furthermore, CS-TA-mediated microenvironment induced an enhanced cell proliferation, chondrogenetic differentiation and cartilaginous ECM accumulation compared to the CMP-TA hydrogel. In particular, we found that the hydrogel with a CMP-TA/CS-TA weight ratio of 3/1 provided a best host tissue-mimetic microenvironment for maintaining chondrocyte phenotype and enhancing chondrogenesis in our experiment range. In addition, *in vivo* testing confirmed that the hydrogels used in this system were well tolerated within a mouse subcutaneous implantation model. We believe the as-prepared injectable biomimetic hydrogels based on polysaccharide hybrids are very promising for the development of scaffolds for CTTE.

## Methods

Materials and detailed experimental methods can be found in Supplementary Information. All experimental protocols reported were approved by Shanghai Jiao Tong University. All animal procedures were carried out in accordance with the approved guidelines and approved by Shanghai Jiao Tong University Committee on Animal Research and Ethic.

## Additional Information

**How to cite this article**: Chen, F. *et al*. An Injectable Enzymatically Crosslinked Carboxymethylated Pullulan/Chondroitin Sulfate Hydrogel for Cartilage Tissue Engineering. *Sci. Rep*. **6**, 20014; doi: 10.1038/srep20014 (2016).

## Supplementary Material

Supplementary Information

## References

[b1] Jin R. . Enzymatically crosslinked dextran-tyramine hydrogels as injectable scaffolds for cartilage tissue engineering. Tissue Eng. Part A. 16, 2429–2440 (2010). 20214454 10.1089/ten.TEA.2009.0764

[b2] Moreira Teixeira L. S. . Self-attaching and cell-attracting *in-situ* forming dextran-tyramine conjugates hydrogels for arthroscopic cartilage repair. Biomaterials. 33, 3164–3174 (2012). 22265787 10.1016/j.biomaterials.2012.01.001

[b3] Jin R. . Chondrogenesis in injectable enzymatically crosslinked heparin/dextran hydrogels. J. Controlled Release. 152, 186 (2011). 10.1016/j.jconrel.2011.01.031 21291927

[b4] Izumikawa T., Sato B. & Kitagawa H. Chondroitin Sulfate Is Indispensable for Pluripotency and Differentiation of Mouse Embryonic Stem Cells. Sci. Rep. 4, 3710 (2014). 24424429 10.1038/srep03701 PMC3892716

[b5] Coburn J. M., Gibson Matthew., Monagle S., Patterson Z. & Elisseeff J. H. Bioinspired nanofibers support chondrogenesis for articular cartilage repair. Proc. Natl. Acad. Sci.USA 109, 10012–10017 (2012). 22665791 10.1073/pnas.1121605109 PMC3382486

[b6] Li Y., Rodrigues J. & Tomas H. Injectable and biodegradable hydrogels: gelation, biodegradation and biomedical applications. Chem. Soc. Rev. 41, 2193–2221 (2012). 22116474 10.1039/c1cs15203c

[b7] Yang J. A., Yeom J., Hwang B. W., Hoffman A. S. & Hahn S. K. *In situ*-forming injectable hydrogels for regenerative medicine. Prog. Polym. Sci. 39, 1973–1986 (2014).

[b8] Yao X., Peng R. & Ding J. Cell-material interactions revealed via material techniques of surface patterning. Adv. Mater. 25, 5257–5286 (2013). 24038153 10.1002/adma.201301762

[b9] Mujeeb A., Miller A. F., Saiani A. & Gough J. E. Self-assembled octapeptide scaffolds for *in vitro* chondrocyte culture. Acta Biomater. 9, 4609–4617 (2013). 22963851 10.1016/j.actbio.2012.08.044

[b10] Balakrishnan B. & Banerjee R. Biopolymer-based hydrogels for cartilage tissue engineering. Chem. Rev. 111, 4453–4474 (2011). 21417222 10.1021/cr100123h

[b11] Rice J. J. . Engineering the regenerative microenvironment with biomaterials. Adv. healthcare mater. 2, 57–71 (2013). 10.1002/adhm.201200197 23184739

[b12] Park K. M. . Synthesis and characterizations of *in situ* cross-linkable gelatin and 4-arm-PPO-PEO hybrid hydrogels via enzymatic reaction for tissue regenerative medicine. Biomacromolecules. 13, 604–611(2012). 22263670 10.1021/bm201712z

[b13] Frith J. E. . An injectable hydrogel incorporating mesenchymal precursor cells and pentosan polysulphate for intervertebral disc regeneration. Biomaterials. 34, 9430–9440 (2013). 24050877 10.1016/j.biomaterials.2013.08.072

[b14] Menzies D. J. . Tailorable cell culture platforms from enzymatically cross-linked multifunctional poly(ethylene glycol)-based hydrogels. Biomacromolecules. 14, 413–423 (2013). 23259935 10.1021/bm301652q

[b15] Ren K., He C., Cheng Y., Li G. & Chen X. Injectable enzymatically crosslinked hydrogels based on a poly(l-glutamic acid) graft copolymer. Polym.Chem. 5, 5069–5076 (2014).

[b16] Balakrishnan B., Joshi N., Jayakrishnan A. & Banerjee R. Self-crosslinked oxidized alginate/gelatin hydrogel as injectable, adhesive biomimetic scaffolds for cartilage regeneration. Acta Biomater. 10, 3650–3663 (2014). 24811827 10.1016/j.actbio.2014.04.031

[b17] Teixeira L. S., Feijen J., van Blitterswijk C. A., Dijkstra P. J. & Karperien M. Enzyme-catalyzed crosslinkable hydrogels: emerging strategies for tissue engineering. Biomaterials. 33, 1281–1290 (2012). 22118821 10.1016/j.biomaterials.2011.10.067

[b18] Portalska K. J. . Boosting angiogenesis and functional vascularization in injectable dextran-hyaluronic acid hydrogels by endothelial-like mesenchymal stromal cells. Tissue Eng. Part A. 20, 819–829 (2014). 24070233 10.1089/ten.TEA.2013.0280

[b19] Jin R. . Enzymatically-crosslinked injectable hydrogels based on biomimetic dextran-hyaluronic acid conjugates for cartilage tissue engineering. Biomaterials. 31, 3103–3113 (2010). 20116847 10.1016/j.biomaterials.2010.01.013

[b20] Lee F., Chung J. E. & Kurisawa M. An injectable enzymatically crosslinked hyaluronic acid–tyramine hydrogel system with independent tuning of mechanical strength and gelation rate. Soft Matter. 4, 880 (2008). 10.1039/b719557e 32907194

[b21] Hou J., Li C., Guan Y., Zhang Y. & Zhu X. X. Enzymatically crosslinked alginate hydrogels with improved adhesion properties. Polym. Chem. 6, 2204–2213 (2015).

[b22] Park K. M., Ko K. S., Joung Y. K., Shin H. & Park K. D. *In situ* cross-linkable gelatin–poly(ethylene glycol) tyramine hydrogel via enzyme-mediated reaction for tissue regenerative medicine. J. Mater. Chem. 21, 13180–13187 (2011).

[b23] Wang L. S. . Modulation of chondrocyte functions and stiffness-dependent cartilage repair using an injectable enzymatically crosslinked hydrogel with tunable mechanical properties. Biomaterials 35, 2207–2217 (2014). 24333028 10.1016/j.biomaterials.2013.11.070

[b24] Jin R., Lin C. & Cao A. Enzyme-mediated fast injectable hydrogels based on chitosan–glycolic acid/tyrosine: preparation, characterization, and chondrocyte culture. Polym. Chem. 5, 391–398 (2014).

[b25] Ren K., He C., Xiao C., Li G. & Chen X. Injectable glycopolypeptide hydrogels as biomimetic scaffolds for cartilage tissue engineering. Biomaterials. 51, 238–249 (2015). 25771014 10.1016/j.biomaterials.2015.02.026

[b26] Kyung M. P., Young M. S., Yoon K. J., Heungsoo S. D. & Ki P. *In Situ* Forming Hydrogels Based on Tyramine Conjugated 4-Arm-PPO-PEO via Enzymatic Oxidative Reaction. Biomacromolecules. 11, 706–712 (2010). 20121075 10.1021/bm9012875

[b27] Autissier A., Le Visage C., Pouzet C., Chaubet F. & Letourneur D. Fabrication of porous polysaccharide based scaffolds using a combined freeze-drying/cross-linking process. Acta Biomater. 6, 3640–3648 (2010). 20215057 10.1016/j.actbio.2010.03.004

[b28] Bae H. . Cell-laden microengineered pullulan methacrylate hydrogels promote cell proliferation and 3D cluster formation. Soft Matter. 7, 1903–1911 (2011). 21415929 10.1039/C0SM00697A PMC3057074

[b29] Singh R. S., Kaur N. & Kennedy J. F. Pullulan and pullulan derivatives as promising biomolecules for drug and gene targeting. Carbohydr. Polym. 123, 190–207 (2015). 25843851 10.1016/j.carbpol.2015.01.032

[b30] Yang X. C., Niu Y. L., Zhao N. N., Mao C. & Xu F. J. A biocleavable pullulan-based vector via ATRP for liver cell-targeting gene delivery. Biomaterials 35, 3873–3884 (2014). 24485791 10.1016/j.biomaterials.2014.01.036

[b31] Victor W. Wong. . Engineered pullulan collagen composite dermal hydrogels improve early cutaneous wound healing. Tissue Eng. part A. 17, 631–664 (2011). 20919949 10.1089/ten.tea.2010.0298 PMC4398002

[b32] Jung Y. S., Park W. & Na K. Temperature-modulated noncovalent interaction controllable complex for the long term delivery of etanercept to treat rheumatoid arthritis. J. Controlled Release. 171, 143–151(2013). 10.1016/j.jconrel.2013.07.012 23880471

[b33] Guerrero J. . The Use of Total Human Bone Marrow Fraction in a Direct Three-Dimensional Expansion Approach for Bone Tissue Engineering Applications: Focus on Angiogenesis and Osteogenesis. Tissue Eng. Part A. 21, 861–874 (2015). 25333855 10.1089/ten.tea.2014.0367 PMC4356225

[b34] Le Visage C. . Mesenchymal stem cell delivery into rat infarcted myocardium using a porous polysaccharide-based scaffold: a quantitative comparison with endocardial injection. Tissue Eng. Part A. 18, 35–44 (2012). 21770864 10.1089/ten.TEA.2011.0053 PMC3626173

[b35] Rustad K. C. . Enhancement of mesenchymal stem cell angiogenic capacity and stemness by a biomimetic hydrogel scaffold. Biomaterials. 33, 80–90 (2012). 21963148 10.1016/j.biomaterials.2011.09.041 PMC3997302

[b36] Lavergne M. . Porous polysaccharide-based scaffolds for human endothelial progenitor cells. Macromol. Biosci. 12, 901–910 (2012). 22696505 10.1002/mabi.201100431

[b37] Shi L., Aid R., Le Visage C. & Chew S. Y. Biomimicking polysaccharide nanofibers promote vascular phenotypes: a potential application for vascular tissue engineering. Macromol. Biosci. 12, 395–401 (2012). 22223225 10.1002/mabi.201100336

[b38] Heymann D. . Pullulan/dextran/nHA Macroporous Composite Beads for Bone Repair in a Femoral Condyle Defect in Rats. PLoS One. 9, e110251 (2014). 25330002 10.1371/journal.pone.0110251 PMC4203774

[b39] Bulman S. E. . Pullulan: a new cytoadhesive for cell mediated cartilage repair. Stem Cell Res. Ther. 6, 34 (2015). 25889571 10.1186/s13287-015-0011-7 PMC4414433

[b40] Bouten P. J. M. . The chemistry of tissue adhesive materials. Prog. Polym. Sci. 39, 1375–1405 (2014).

[b41] Varghese S. . Chondroitin sulfate based niches for chondrogenic differentiation of mesenchymal stem cells. Matrix. Biol. 27, 12–21 (2008). 17689060 10.1016/j.matbio.2007.07.002

[b42] Wang D. A. . Multifunctional chondroitin sulphate for cartilage tissue-biomaterial integration. Nat. Mater. 6, 385–392 (2007). 17435762 10.1038/nmat1890 PMC8128046

[b43] Chang K. Y., Cheng L. W., Ho G. H., Huang Y. P. & Lee Y. D. Fabrication and characterization of poly(gamma-glutamic acid)-graft-chondroitin sulfate/polycaprolactone porous scaffolds for cartilage tissue engineering. Acta Biomater. 5, 1937–1947 (2009). 19282262 10.1016/j.actbio.2009.02.002

[b44] Liao J., Qu Y., Chu B., Zhang X. & Qian Z. Biodegradable CSMA/PECA/Graphene Porous Hybrid Scaffold for Cartilage Tissue Engineering. Sci. Rep. 5, 9879 (2015). 25961959 10.1038/srep09879 PMC4426702

[b45] Mocanua G., Mihaia D., Picton L., LeCerfb D. & Mullerb G. Associative pullulan gels and their interaction with biological active substances. J. Controlled Release. 83, 41–51 (2002). 10.1016/s0168-3659(02)00169-4 12220837

[b46] Weng L. H., Chen X. M. & Chen W. Rheological characterization of *in situ* crosslinkable hydrogels formulated from oxidized dextran and N-carboxyethyl chitosan. Biomacromolecules. 8, 1109–1115 (2007). 17358076 10.1021/bm0610065 PMC2572577

[b47] Ingavle G. C., Frei A. W., Gehrke S. H. & Detamore M. S. Incorporation of aggrecan in interpenetrating network hydrogels to improve cellular performance for cartilage tissue engineering. Tissue Eng. Part A. 19, 1349–1359 (2013). 23379843 10.1089/ten.tea.2012.0160 PMC3638541

[b48] Levett P. A. . A biomimetic extracellular matrix for cartilage tissue engineering centered on photocurable gelatin, hyaluronic acid and chondroitin sulfate. Acta Biomater. 10, 214–223 (2014). 24140603 10.1016/j.actbio.2013.10.005

[b49] Lim J. J. & Temenoff J. S. The effect of desulfation of chondroitin sulfate on interactions with positively charged growth factors and upregulation of cartilaginous markers in encapsulated MSCs. Biomaterials. 34, 5007–5018 (2013). 23570717 10.1016/j.biomaterials.2013.03.037 PMC3671883

[b50] Bryant S. J., Arthur J. A. & Anseth K. S. Incorporation of tissue-specific molecules alters chondrocyte metabolism and gene expression in photocrosslinked hydrogels. Acta Biomater. 1, 243–252 (2005). 16701801 10.1016/j.actbio.2004.11.003

[b51] DeLise A. M., Fischer L. & Tuan R. S. Cellular interactions and signaling in cartilage development. Osteoarthritis Cartilage. 8, 309–334 (2000). 10966838 10.1053/joca.1999.0306

[b52] Villanueva I., Gladem S. K., Kessler J. & Bryant S. J. Dynamic loading stimulates chondrocyte biosynthesis when encapsulated in charged hydrogels prepared from poly(ethylene glycol) and chondroitin sulfate. Matrix. Biol. 29, 51–62 (2010). 19720146 10.1016/j.matbio.2009.08.004 PMC2914691

[b53] Villanueva I., Bishop N. L. & Bryant S. J. Medium osmolarity and pericellular matrix development improves chondrocyte survival when photoencapsulated in poly(ethylene glycol) hydrogels at low densities. Tissue Eng. part A. 15, 3037–3048 (2009). 19331581 10.1089/ten.tea.2009.0001 PMC2792074

