# Supplementary material for: An Injectable Enzymatically Crosslinked Carboxymethylated Pullulan/Chondroitin Sulfate Hydrogel for Cartilage Tissue Engineering
Source: Sci Rep. 2016 Jan 28;6:20014. doi: 10.1038/srep20014 (PMC4730219; doi:10.1038/srep20014)
Supplement: Supplementary Information [file srep20014-s1.pdf]

Supplementary Information

An injectable enzymatically crosslinked carboxymethylated pullulan/chondroitin sulfate hydrogel for cartilage tissue engineering

Feng Chen, Songrui Yu, Bing Liu, Yunzhou Ni, Chunyang Yu, Yue Su, Xinyuan Zhu,

Xiaowei Yu, Yongfeng Zhou and Deyue Yan

A) Supplemental results and discussion

As shown in Fig. S3A, the gelation time of CMP-TA/CS-TA (1/1, w/w) hydrogel remarkably decreased from 640 to 33 s as HRP concentration increased from 0.1 to 4.8 unit/mL at constant polymer concentration (5 wt. %) and H_2O_2 concentration (1mM). This result could be explained by the fact that the generation rate of the phenoxy free radical increased in the coupling reaction as HRP concentration increased.[1] In contrast, the gelation time increased as H_2O_2 concentration increased (Fig. S3B). The gelation time slightly increased from 105 to 169 s as H_2O_2 concentration increased from 1 to 10 mM, indicating that gelation rate had low dependencies of H_2O_2 concentration. Further, a rapid rise in gelation time was observed when H_2O_2 concentration was increased from 10 to 25 mM. These results occurred because HRP adopted an inactivated configuration due to excess oxidation in the presence of excess H_2O_2[1-2]. The influence of HRP and H_2O_2 concentrations on the gelation time was consistent with some polymer derivatives[3]. The gelation time decreased from 127 to 65 s when polymer concentration increased from 3 to 10 % at fixed HRP concentration of 0.6 unit/mL and H_2O_2 concentration of 1mM ($p < 0.01$) (Fig. S3C). This result could be explained by the fact that an increase in TA moiety resulted in an increased chance for cross-linking between each phenol substrate.

Fig. S4 showed the effect of HRP concentration on the rheological behavior of CMP-TA/CS-TA (1/1) hydrogel at fixed polymer concentration of 5 wt. % and H_2O_2 concentration of 1mM. When increasing HRP concentration from 0.1 to 1.2 unit/mL, the G' values increased from 18 to 430 Pa. Interestingly, the G' remained almost constant at or above 1.2 unit/mL (Fig. S4A). From the frequency sweep experiments, it was found that, with the exception

of the hydrogel formed using 0.1 unit/mL HRP, the rest of hydrogels was elastic and the G' was not dependent on the frequency (Fig. S4B). In addition, the fracture stress of the hydrogel of the lowest HRP concentration was about 125 Pa, however, the fracture stress values of the rest of hydrogels were about 400 Pa in the stress sweeps (Fig. S4D). The above results confirmed that the hydrogel of the lowest HRP concentration had a weakest mechanical strength. This should be due to the fact that , though HRP plays a role as catalyst in this system, excess H_2O_2 may cause excessive oxidation and inactivation of HRP and thereby give rise to decreased crosslinking efficiency. The effect of H_2O_2 concentration on the rheological behavior of CMP-TA/CS-TA (1/1) hydrogel at fixed polymer concentration of 5 wt. % and HRP concentration of 0.6 unit/mL was investigated (Fig. S5). The G' values remarkably increased from about 299 to 1263 Pa with H_2O_2 concentration increase from 1 mM to 5 mM suggesting higher crosslinking density was achieved when H_2O_2 concentration increased. However, further increase of H_2O_2 concentration resulted in a decline of G', which was likely due to the deactivation of HRP by an excess amount of H_2O_2 as previously mentioned[3e] (Fig. S5A). For the frequency sweep, except for the H_2O_2 concentration of 25 mM, the G' of the rest of hydrogels remained independent of the frequency (Fig. S5B), demonstrating that these hydrogels displayed a predominantly elastic behavior. The fracture stress reached to a maximum at H_2O_2 concentration of 5 mM in the stress sweeps (Fig. S5D). Moreover, the variation tendency of the fracture stress of the hydrogels was consistent with the G' in time sweeps. In contrast to HRP concentration, the stiffness of hydrogel very depended on H_2O_2 concentration. These results were in good agreement with previous reports of the hydrogel systems using the same enzymatic oxidation reaction[3b, 3d]. Fig. S6 shown that polymer concentration had an influence on the rheological behavior of CMP-TA/CS-TA (1/1) hydrogel at fixed HRP concentration of 0.6 unit/mL and H_2O_2 concentration of 1 mM. It was noteworthy that the increase in polymer concentration led to higher G' and fracture stress as a result of higher amounts of TA moieties participating in the crosslinking reaction (Fig. S6A and D). Furthermore, the G' was almost insensitive to the oscillatory frequency

within the range of 3-10% (Fig. S6B). The impacts of increasing polymer concentration align with those reported by Jin et al. and Menzies et al[3b, 4] . Additionally, the G' values were always larger than the G″ values indicating that all hydrogels display a predominantly elastic behavior in the frequency sweep tests (Fig. S4C, S5C and S6C).

The ESR behavior of CMP-TA/CS-TA hydrogels (1/1) prepared with different concentrations of HRP at fixed H_2O_2 concentration of 5 mM and polymer concentration of 5 wt. % was shown in Fig. S7A. The ESR slightly decreased from 30 to 27 as HRP concentration increased from 0.6 to 2.4unit/mL. Further, when HRP concentration at or above 2.4 unit/mL, the ESR almost remained about 27. These results clearly indicated that HRP concentration had a minor influence on the ESR of the hydrogels. The effect of H_2O_2 concentration on the ESR and the degradation behavior of CMP-TA/CS-TA (1/1) hydrogel at fixed polymer concentration (5 wt. %) and HRP concentration (4.8 unit/mL) was displayed in Fig. S7B and C, respectively. In the examined range of H_2O_2 concentration, the ESR ranged from 27 to 30. Additionally, the hydrogels prepared with H_2O_2 concentrations of 1 and 25 mM were completely degraded after 6 day. After 21 day degradation, the weight remaining ratios of the hydrogels prepared with H_2O_2 concentration of 2, 5, and 10 mM were 45 %, 61 % and 41 %, respectively. When H_2O_2 concentration increased from 1 to 5 mM, the ESR was lower and the hydrogels were degraded slower, suggesting that the cross-linking density increased at higher H_2O_2 concentration. However, further increased H_2O_2 concentration resulted in the higher ESR and faster degradation rate, which might be attribute to excessive oxidation of HRP caused by H_2O_2 and resulted in a decreased efficiency of the cross-linking reaction as previously reported[3d].

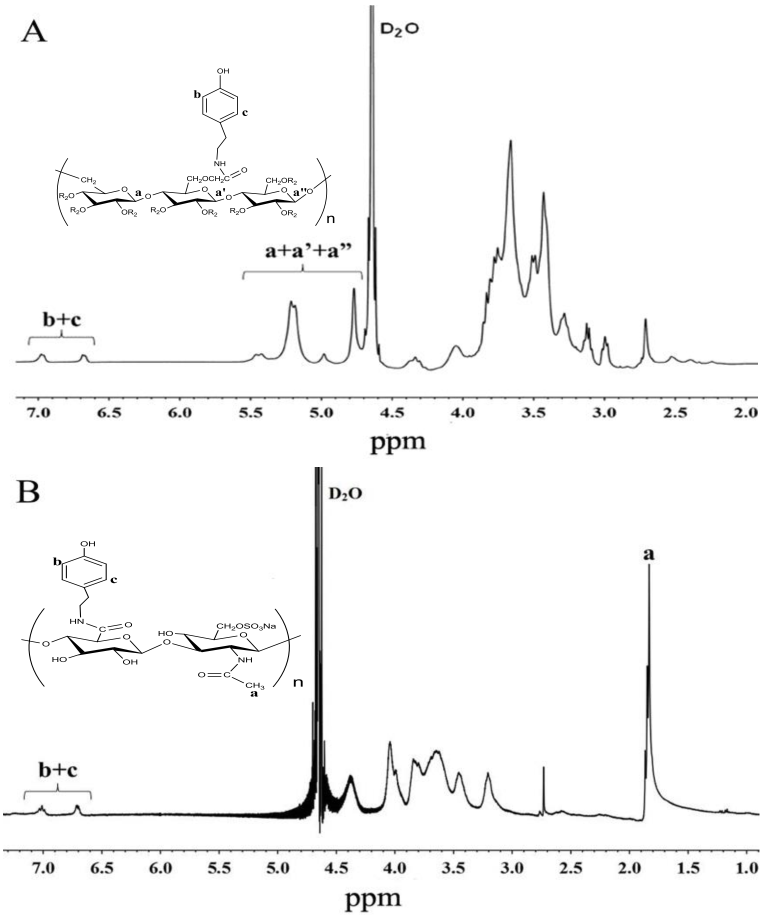

Figure S1: ^{1}H NMR spectra of CMP-TA (A) and CS-TA (B) in D_2O. The DS of CMP-TA (the number of TA molecules per 100 anhydroglucose units of CMP) was calculated by comparing the integrals of signals at δ 4.8–5.6 (CMP anomeric protons) and δ=6.5–7.0 (TA aromatic protons). The DS of CS-TA (the number of TA molecules per 100 repeating units of CS) was calculated by comparing the integrals of signals at δ=1.8 (acetamide methyl protons of CS) and δ=6.5–7.0.

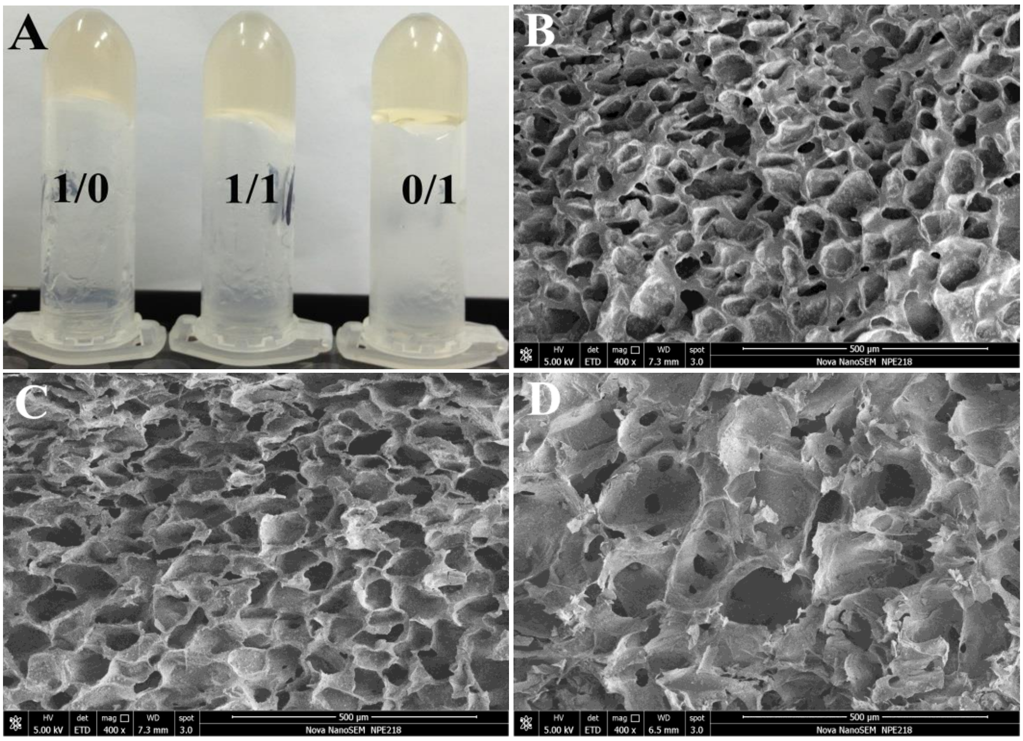

Figure S2: The optical (A) and SEM images of CMP-TA/CS-TA hydrogels as a function of CMP-TA/CS-TA weight ratio, and the final concentrations of polymer, HRP and H_2O_2 were kept at 5 wt. %, 0.6 units/mL and 1 mM. Cross-sectional morphologies of the hydrogels: 1/0 (B), 1/1 (C) and 0/1(D).

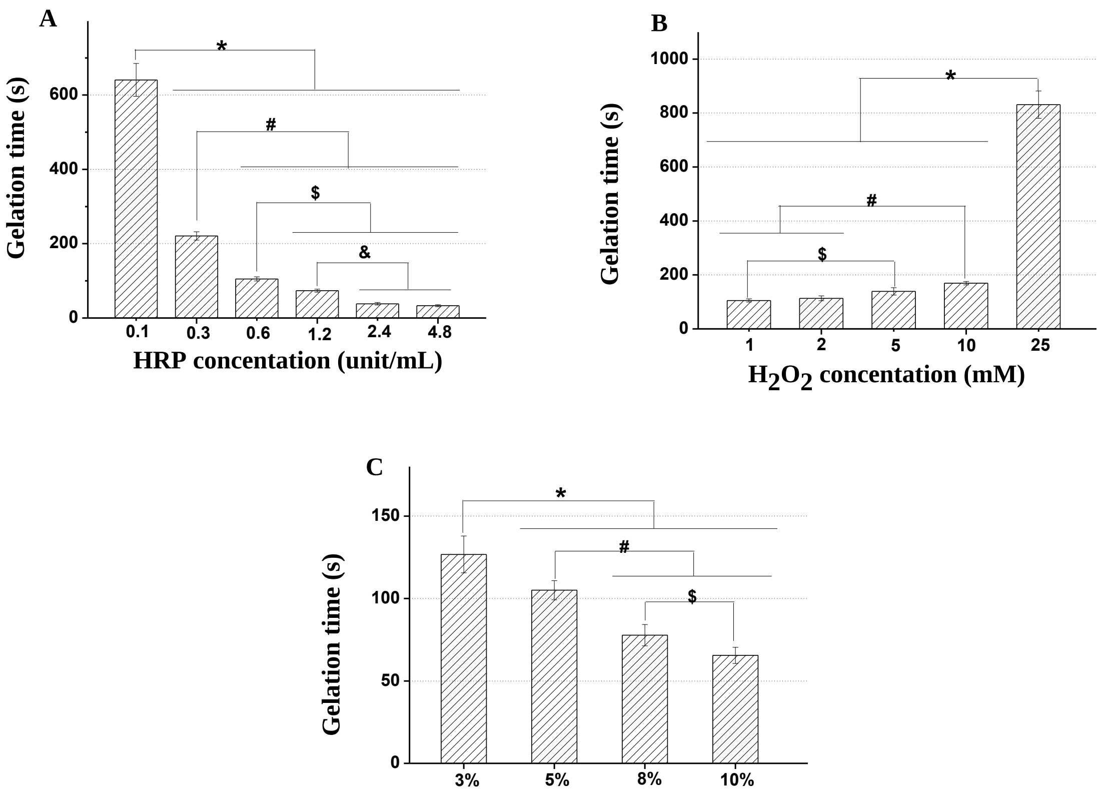

Figure S3: (A) Gelation time of the hydrogels (1/1, w/w) as a function of HRP concentration, and the final concentrations of polymer and H_2O_2 were kept at 5 wt. % and 1mM, respectively (n=8, $p < 0.01$). (B) Gelation time of the hydrogels (1/1, w/w) as a function of H_2O_2 concentration, and the final concentrations of polymer and HRP were kept at 5 wt. % and 0.6 units/mL, respectively (n=8, $p < 0.01$). (C) Gelation time of the hydrogels (1/1, w/w) as a function of polymer concentration, and the final concentrations of HRP and H_2O_2 were kept at 0.6 units/mL and 1mM, respectively (n=8, $p < 0.01$).

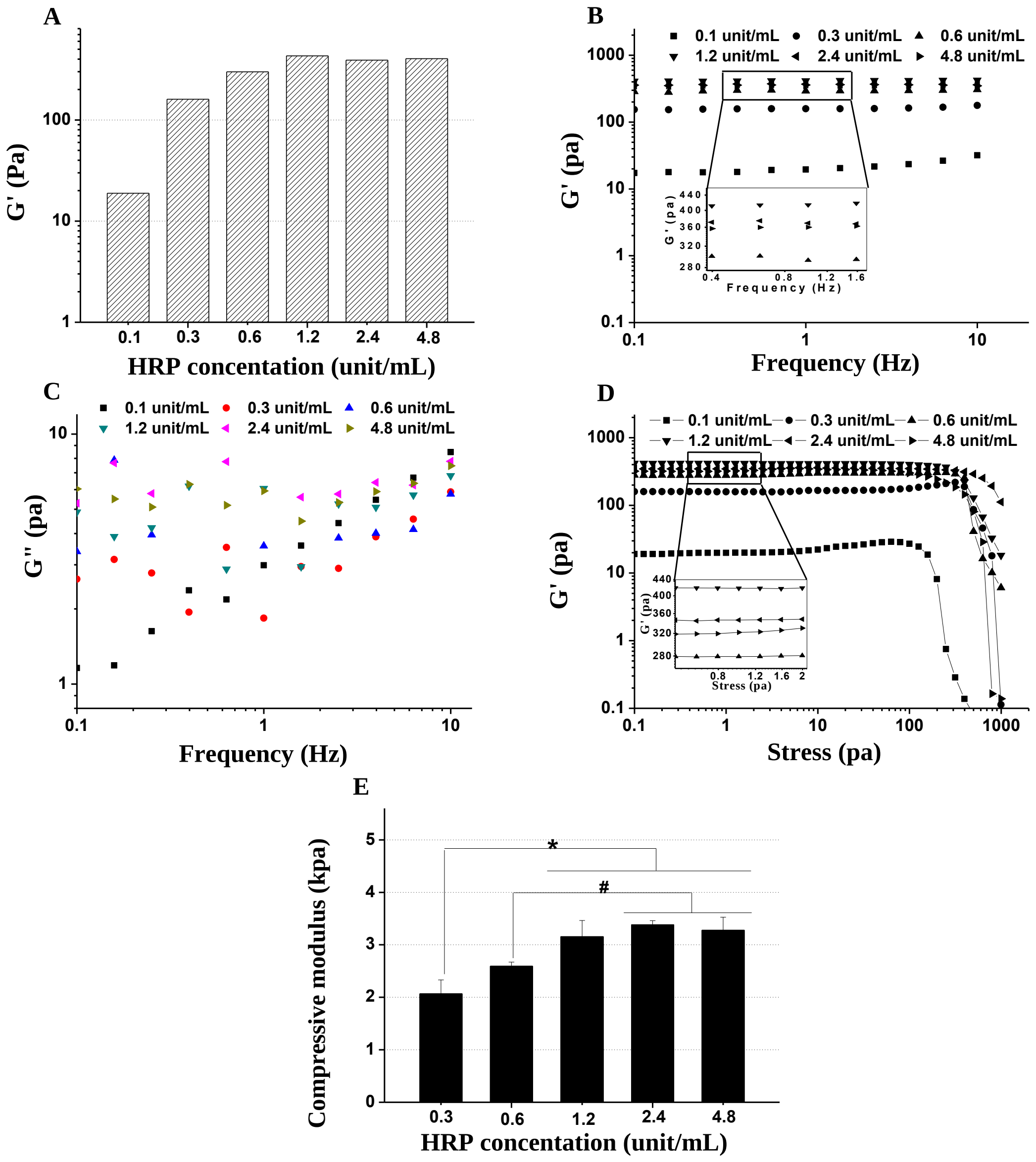

Figure S4: Storage moduli (G') (A), Frequency sweep (B, C), Stress sweep (D) and Compressive modulus (n=3, p < 0.01) (E) of CMP-TA/CS-TA hydrogels (1/1, w/w) as a function of HRP concentration, and the final concentrations of polymer and H_2O_2 were kept at 5 wt. % and 1mM, respectively.

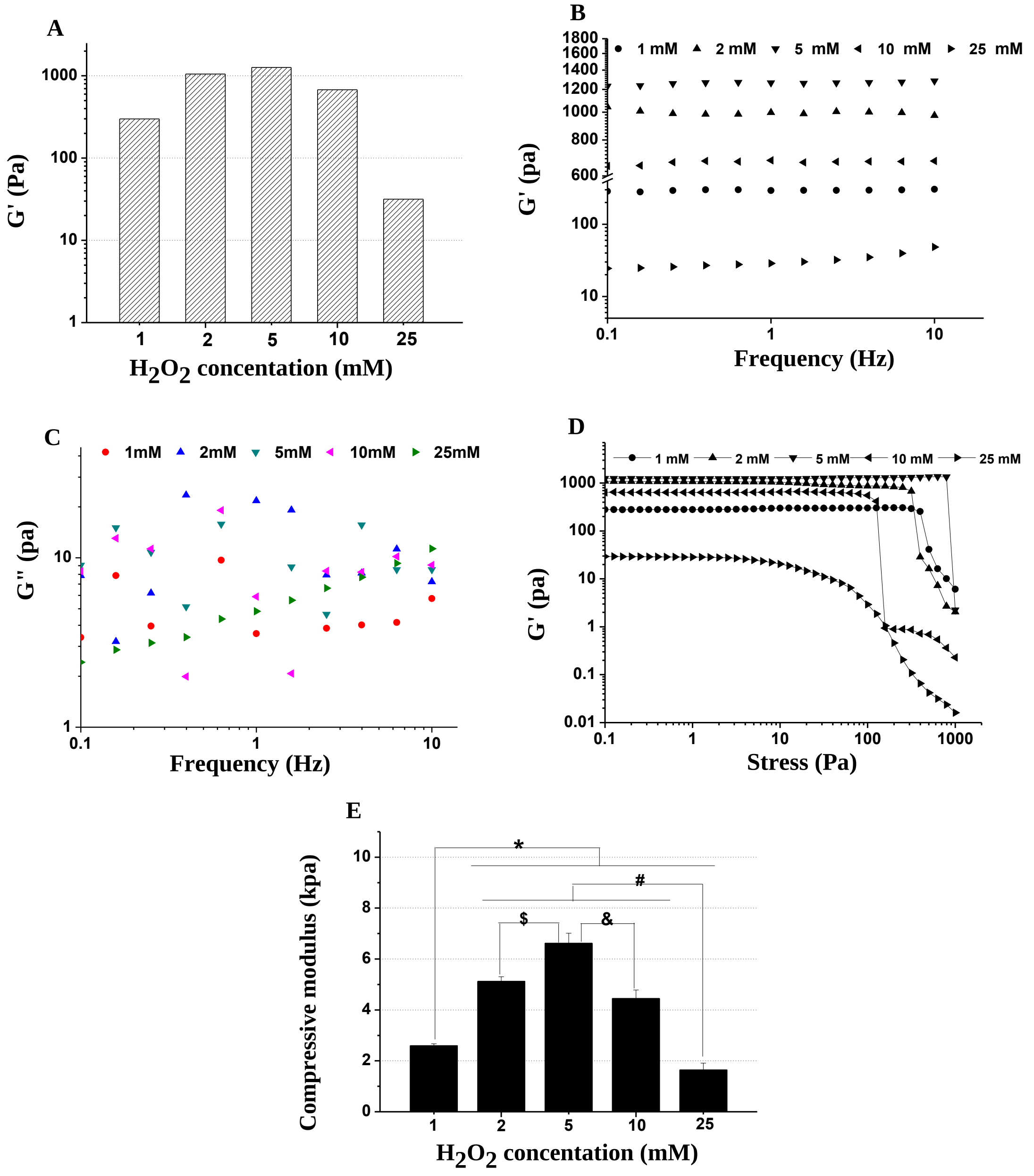

Figure S5: Storage moduli (G') (A), Frequency sweep (B, C), Stress sweep (D) and Compressive modulus (n=3, $p < 0.01$) (E) of CMP-TA/CS-TA hydrogels (1/1, w/w) as a function of H_2O_2 concentration, and the final concentrations of polymer and HRP were kept at 5 wt. % and 0.6 units/mL, respectively.

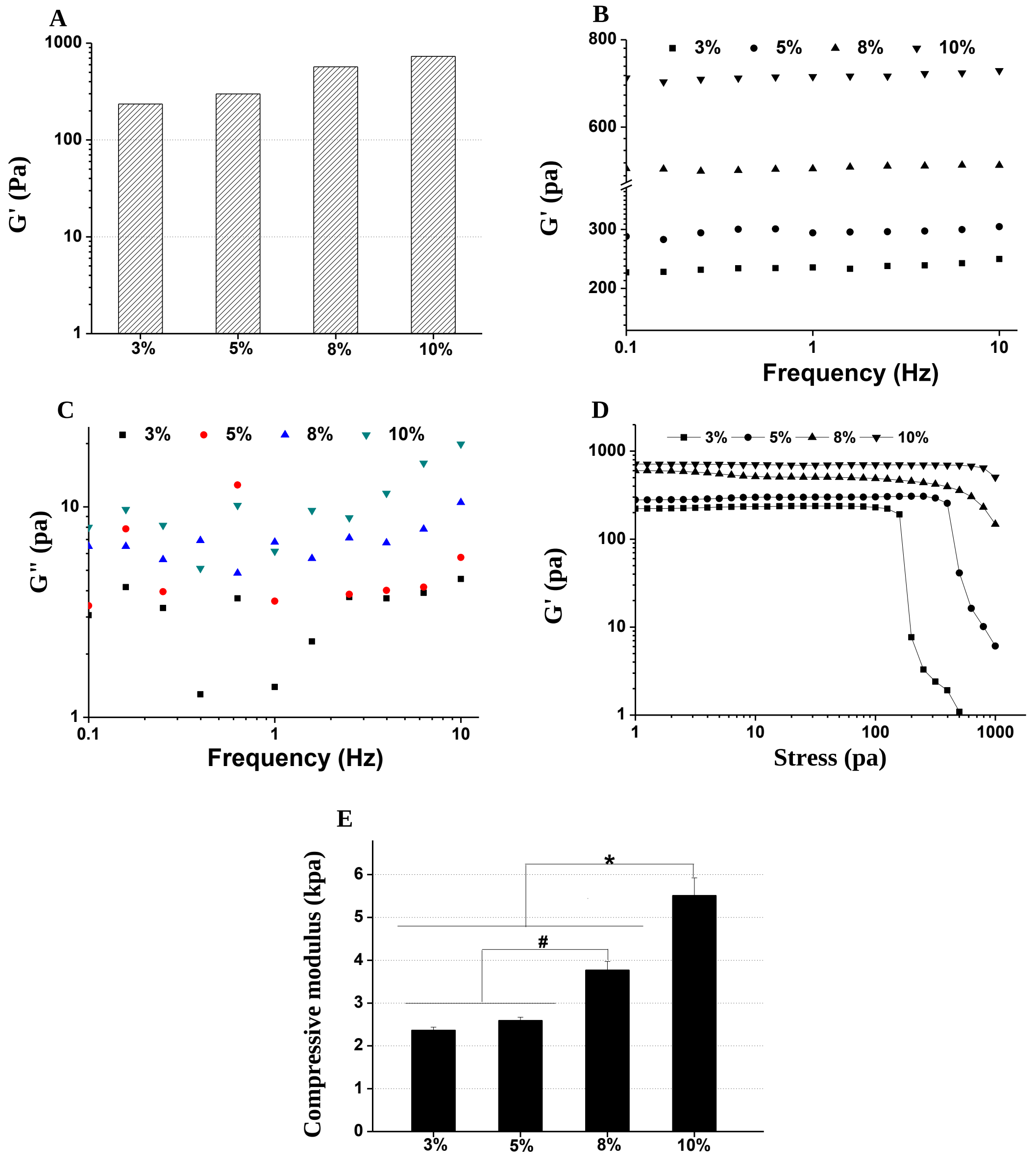

Figure S6: Storage moduli (G') (A), Frequency sweep (B, C), Stress sweep (D) and Compressive modulus (n=3, $p < 0.01$) (E) of CMP-TA/CS-TA hydrogels (1/1, w/w) as a function of polymer concentration, and the final concentrations of HRP and H_2O_2 were kept at 0.6 units/mL and 1mM, respectively.

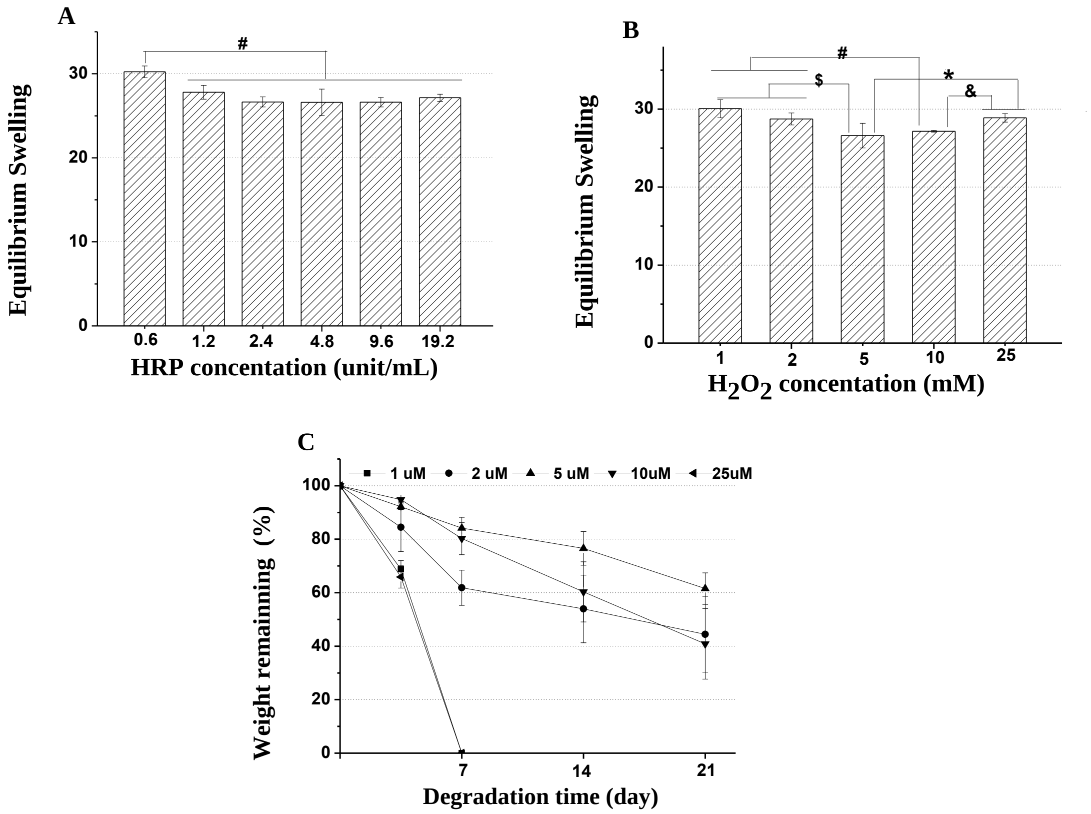

Figure S7: (A) The ESR of CMP-TA/CS-TA hydrogels (1/1, w/w) as a function of HRP concentration, and the final concentrations of polymer and H_2O_2 were kept at 5 wt. % and 5mM, respectively (n=6, $p < 0.01$). (B) The ESR of CMP-TA/CS-TA hydrogels (1/1, w/w) as a function of H_2O_2 concentration, and the final concentrations of polymer and HRP were kept at 5 wt. % and 4.8 units/mL, respectively (n=6, $p < 0.01$). (C) Degradation of the hydrogels (1/1, w/w) as a function of H_2O_2 concentration, and the final concentrations of polymer and HRP were kept at 5 wt. % and 4.8 units/mL, respectively (n =6).

Table S1 The equilibrium swelling ratio (ESR) of CMP-TA/CS-TA hydrogels

Weight ratio	Medium		
	PBS	H_2O	Hanks'
1/0	13.70±0.89	15.12±1.16	12.98±0.57
3/1	19.20±1.06	22.78±0.65*	20.37±1.54
1/1	26.60±1.58	34. 20±1.30*	27.84±1.63
1/3	32.82±0.41	40.54 ±1.22*	33.51±1.15
0/1	41.71±1.74	49.03 ±1.52*	39.86±1.96

Table S1 shows the ESR of CMP-TA/CS-TA hydrogels as a function of CMP-TA/CS-TA weight ratio in the PBS, pure water and tissue fluid-like solution, and the final concentrations of polymer, HRP and H_2O_2 were kept at 5 wt. % , 4.8 units/mL and 5 mM, respectively (n=6, * $p < 0.01$). Irrespective of the variation of the test medium, all the ESR values of the CMP-TA/CS-TA hydrogels significantly increased with increasing the CS-TA content ($p < 0.01$). The results could be attributed to both the lower crosslinking density and the higher hydrophilic property of the gels due to the introduction of more highly negatively charged CS-TA chains. In addition, expect for the pure CMP-TA hydrogel, the ESR values of the rest of hydrogel in the pure water were remarkably higher than those in the PBS or the tissue fluid-like solution ($p < 0.01$), while there are no big difference when compared the ESR values in the PBS solution with those in the tissue fluid-like solution. It is due to the strong shielding effect of the polyelectrolyte gels with counter ions in PBS and tissue fluid-like solution, while it is not important in pure water due to the deficiency of ions inside.

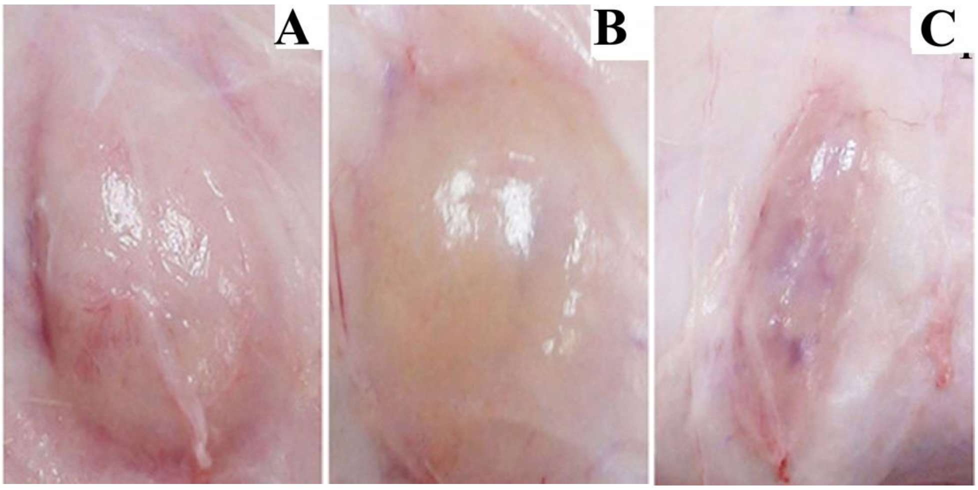

Figure S8: The implanted CMP-TA/CS-TA hydrogels of 1/0 (A), 3/1 (B) and 1/1 (C) on the backside of SD rats: the cutaneous tissue containing the implanted hydrogels was analyzed after subcutaneous implanting for 4 weeks. M represented residue of the implant.

Table S2 The primers designed for RT-PCR

Gene	Primer Sequence (5'-3')	Size
**Type Ⅰ**	Sense GAAGACATCCCACCAGTCACCT	154
**Collagen**	Antisence CGCTGGGACAGTTCTTGATTTC	
**Type Ⅱ**	Sense TGGGCAGAGGTATAATGATAAGGA	101
**Collagen**	Antisence CTTCACAGATTATGTCGTCGCAG	
**Aggrecan**	Sense TGGGAAAGAAGACTTGGCTGG	232
	Antisence CTCCACCAATGTAGTATCCACGA	
**β- actin**	Sense CATCGTCCACCGCAAATGCTTCT	217
	Antisence CGACTGCTGTCACCTTCACCGTTC	

B) Supplementary Experimental

Materials: Pullulan, CS, Tyramine hydrochloride (TA HCI) and 1-Hydroxybenzotriazole monohydrate (HOBt) were purchased from TCI (Shanghai) development Co., Ltd. N-ethyl-N′-(3-dimethylaminopropyl) carbodiimide hydrochloride (EDC HCI) was purchased from Adamas. H_2O_2 was obtained from Alfa Aesar. Chloroacetic acid sodium ($ClCH_2COONa$) was purchased from J&K technology co., Ltd (China). 4-morpholino ethanesulfonic acid (MES), N-hydroxysuccinimide (NHS), Horseradish peroxidase (HRP, 250-330 pyrogallol units/mg solid) and

Proteinase K (from Tritirachium album, ≥30 units/mg protein) were obtained from Aldrich-Sigma. All other solvents were purchased from Sinopharm Chemical Reagent Co., Ltd (China). Phosphate buffered saline (PBS, pH=7.4, 0.01 M) was prepared by dissolving 1.145 g of di-sodium hydrogen phosphate, 0.2 g of di-potassium hydrogen phosphate, 0.2 g of potassium chloride and 8 g of sodium chloride in 1 L distilled water. Deionized water was employed throughout.

Synthesis and characterization of carboxymethyl pullulan-tyramine and chondroitin sulfate-tyramine: Carboxymethyl pullulan (CMP) was synthesized beforehand by the reaction of pullulan with $ClCH_3COONa$ in the presence of sodium hydroxide as previously described method[5]. CMP-TA and CS-TA were synthesized by the coupling reaction of amine groups of TA to carboxylic acid groups of CMP and CS using EDC/NHS activation.[4, 6] The degree of substitution (DS) and molecular weight of CMP-TA and CS-TA were analyzed using ^1H NMR (400 MHz, Varian) and GPC (Perkin Elmer), respectively.

Hydrogel formation, morphological studies and the gelation time: CMP-TA/CS-TA hydrogels were prepared in vials at 37 ^oC. In a typical procedure, CMP-TA solution (400 µL, 6.25 wt. %) was mixed with HRP (50 µL, 6 Unit/mL) and H_2O_2 solution (50 µL, 0.01 mM/mL) and gently vortexed. All solutions were dissolved in PBS (pH=7.4, 0.01M). The final concentrations of polymer, HRP and H_2O_2 were 5 wt. %, 0.6 Unit/mL and 1 mM, respectively. The CMP-TA/CS-TA hydrogels at different weight ratios (3/1, 1/1, 1/3 or 0/1) were prepared in the same manner. The internal microstructure of the hydrogels was characterized by scanning electron microscopy (SEM) (PEI, USA) at a voltage of 5 kV. The samples were lyophilized, cross-sectioned and sputter coated with gold before analysis. Additionally, the gelation time of hydrogel was determined using the vial tilting methods. [3d]

Rheological analysis: All rheological measurements were performed with an AR-G2, (TA Instruments, USA) using parallel plates configuration at 37 ^oC. For time sweeping test, the measurement was allowed to proceed at 1 Hz frequency and 2 % strain until the storage moduli (G') reached a plateau value. In a frequency sweeping test,

the polymer sample was maintained between the parallel plates with a gap of 1 mm for 60 minutes and then subjected to frequency scanning from 0.1 to 10 Hz at 2 % strain. Similarly, a stress sweep was performed from 0.1 to 1000 Pa oscillatory stress at 1 Hz frequency.

Compressive modulus: The CMP-TA/CS-TA hydrogels were formed in 24-well plates and left to cross-link for 6 h. The known sizes (height and diameter) of cylindrical hydrogels were compressed to rupture on a Texture Analyser (TA. XT plus, Stable Micro Systems, UK) with a cylinder load of 1 g moving at 0.5 mm/s to plot the force (Pa) as a function of strain. As the initial surface of the cylindrical hydrogel sample was not perfectly flat, the compressive modulus was calculated as the slope of the curve between 5 % and 25 % strain.

Equilibrium swelling ratio: The freeze-dried CMP-TA/CS-TA hydrogels were accurately weighted (W_w), and then immersed in PBS (pH=7.4, 0.01M) at 37 ^oC for 36 h until equilibrium of swelling had been reached. The swollen hydrogels were removed and immediately weighed (W_s) after the excess of water lying on the surfaces was absorbed with filter paper. The equilibrium swelling ratio (ESR) was expressed as (W_s –W_w)/W_w. The equilibrium swelling behaviors in pure water, PBS and tissue fluid-like solution (Hanks' Balanced Salt Solution, H9394, Sigma) were summarized in Table S1.

In vitro degradation: The CMP-TA/CS-TA hydrogels were by lyophilized and accurately recorded initial weight (W_i), and then incubated with PBS (pH=7.4, 0.01M) at 37 ^oC in a shaking water bath (approx. 50 rpm). At specified time intervals, the hydrogels were removed, lyophilized and weighed (W_d), and then fresh media were added to each samples. Percentage weight remaining was calculated by (W_d/ W_i) ×100 %.

Cell isolation and culture: Isolation and culture of porcine auricular chondrocytes were performed as previously described.[7] The chondrocytes were cultured at 37 ^oC with 95 % humidity and 5% CO_2 in chondrocyte specific medium [DMEM with 10 % FBS, 1 % antibiotic-antimycotic (Gibco), 5 μg/mL plasmocin™ treatment and 5 ng/mL recombinant Human FGF basic (146 aa, R&D System)].

Cell encapsulation: Polymer solutions of CMP-TA/CS-TA at different weight ratios (1/0, 3/1 or 1/1) were prepared in the chondrocyte specific medium. HRP and H_2O_2 solutions were made in PBS (pH=7.4, 0.01M, sterilization). Subsequently, chondrocytes were incorporated into the hydrogels using the same procedure as for the gel formation in the absence of cells. The final polymer, HRP and H_2O_2 concentrations were 5wt. %, 4.8 Unit/mL and 5 mM, respectively. The cell seeding density was about 5.0×10^6 cells/mL. After gelation, the cell-laden constructs were cultured in chondrocyte specific medium at 37 ^{o}C in a humidified atmosphere containing 5 % CO_2 for 14 days, and the medium was replaced every two days.

Cell viability and proliferation: We used the Live/Dead viability assay which assesses cell viability. The cell-laden constructs were rinsed with PBS, and incubated in a solution of 2 mM calcein AM (Sigma) and 2 mM ethidium homodimer-1 (EH-1, Sigma) for 20 min at 37 ℃, then visualized using a confocal laser scanning microscope (CLSM, Nikcon). Living cells green and the nuclei of dead cells red fluorescent. To evaluate cell proliferation, quantification of DNA was performed. DNA content of cell-laden constructs was done using the AccuBlue ^{TM} High Sensitivity dsDNA Quantitation Kits (Biotium), according to the manufacturer's instructions.

Quantitative Real-time PCR analysis: Total RNA was extracted from the cell-laden constructs using Trizol reagent (Sigma) following the standard protocols. Subsequently, RNA was reverse transcribed into cDNA using revert aid first strand cDNA Synthesis Kit (Thermo) according to the manufacturer's recommendations. Quantitative Real-time PCR (RT-PCR) was performed using Fast Start Universal SYBR Green Master (Roch) in a 7300 RT-PCR System (ABI), with β -actin as reference genes. The expression levels of targets gene were then calculated as $2^{-\Delta\Delta t}$ as previously described. [8] The primer sequences were listed in **Table S2**.

Matrix production: The secretion of collagen type II and aggrecan by chondrocytes was evaluated by immunofluorescent staining and Western-blot (WB) as previously reported.[9] In addition, total collagen content was determined using the hydroxyproline assay in which hydroxyproline makes up 12.5 % of collagen as previously

reported.[7] Samples were extrapolated against hydroxyproline standards between 0 and 200 μg/mL. All values were corrected for the background staining of gels without cells and normalized to the dry gel mass or DNA content.

In vivo subcutaneous implant of the hydrogels: A mouse subcutaneous implant model was used to evaluate the tissue compatibility. Following 4 weeks of implantation, SD rats were humanely euthanized via carbon dioxide inhalation. Retrieved implants with surrounding tissues were fixed in 10 % paraformaldehyde solution. The fixed samples were then dehydrated, embedded in paraffin and stained with hematoxylin and eosin (H&E). All animals received humane care in compliance with protocols approved by Shanghai Jiao Tong University Animal Research Committee.

Statistical analysis: Data was expressed as mean ±standard deviations (SD). Statistical significance was determined by single- factor analysis of variance followed by Tukey's post hoc analysis. Analysis was performed using the SPSS 13.0 statistical software. Statistical significance was set at $p < 0.01$ or $p < 0.05$.

References

1. Park, K. M., Ko, K. S., Joung, Y. K., Shin, H., Park, K. D. In situ cross-linkable gelatin–poly(ethylene glycol) tyramine hydrogel via enzyme-mediated reaction for tissue regenerative medicine. *J. Mater. Chem.* **21,** 13180-13187 (2011).

2. Jin, R. Lin, C. Cao, A. Enzyme-mediated fast injectable hydrogels based on chitosan–glycolic acid/tyrosine: preparation, characterization, and chondrocyte culture. *Polym. Chem.* **5,** 391-398 (2014).

3. (a) Park, K. M. et al. Synthesis and characterizations of in situ cross-linkable gelatin and 4-arm-PPO-PEO hybrid hydrogels via enzymatic reaction for tissue regenerative medicine. *Biomacromolecules.* **13,** 604-611(2012). (b) Menzies, D. J. et al. Tailorable cell culture platforms from enzymatically cross-linked multifunctional poly(ethylene glycol)-based hydrogels. *Biomacromolecules.***14,** 413-423 (2013). (c) Ren, K., He,

C., Cheng, Y., Li, G., Chen, X. Injectable enzymatically crosslinked hydrogels based on a poly(l-glutamic acid) graft copolymer. *Polym. Chem.* **5,** 5069-5076 (2014). (d) Lee, F., Chung, J. E., Kurisawa, M. An injectable enzymatically crosslinked hyaluronic acid–tyramine hydrogel system with independent tuning of mechanical strength and gelation rate. *Soft Matter.* **4,** 880 (2008). (e) Frith, J. E. et al. An injectable hydrogel incorporating mesenchymal precursor cells and pentosan polysulphate for intervertebral disc regeneration. *Biomaterials.* **34,** 9430-9440 (2013).

4. Jin, R. et al. Enzymatically-crosslinked injectable hydrogels based on biomimetic dextran-hyaluronic acid conjugates for cartilage tissue engineering. *Biomaterials***. 31,** 3103-3113 (2010).
5. Mocanua, G., Mihaia, D., Picton, L., LeCerfb, D., Mullerb, G. Associative pullulan gels and their interaction with biological active substances. *J. Controlled Release.* **83,** 41-51 (2002).
6. Jin, R. et al. Enzymatically crosslinked dextran-tyramine hydrogels as injectable scaffolds for cartilage tissue engineering. *Tissue Eng.Part A.* **16,** 2429-2440 (2010).
7. (a) Jin, R. et al. Chondrogenesis in injectable enzymatically crosslinked heparin/dextran hydrogels. *J. Controlled Release.* **152**, 186 (2011)**.** (b) Wang, L. S., Chung, J. E., Chan, P. P., Kurisawa, M. Injectable biodegradable hydrogels with tunable mechanical properties for the stimulation of neurogenesic differentiation of human mesenchymal stem cells in 3D culture. *Biomaterials.* **31,** 1148-1157 (2010).
8. Levett, P. A. et al. A biomimetic extracellular matrix for cartilage tissue engineering centered on photocurable gelatin, hyaluronic acid and chondroitin sulfate. *Acta Biomater***. 10,** 214-223 (2014).
9. (a) Balakrishnan, B., Joshi, N., Jayakrishnan, A., Banerjee, R. Self-crosslinked oxidized alginate/gelatin hydrogel as injectable, adhesive biomimetic scaffolds for cartilage regeneration. *Acta Biomater.* **10,** 3650-3663 (2014). (b) Lai, J. H., Kajiyama, G., Smith, R. L., Maloney, W., Yang, F. Stem cells catalyze cartilage formation by neonatal articular chondrocytes in 3D biomimetic hydrogels. *Sci. Rep.* **3,** 3553 (2013).
